# Potashchelins, a Suite of Lipid Siderophores Bearing Both L-*threo* and L-*erythro* Beta-Hydroxyaspartic Acids, Acquired From the Potash-Salt-Ore-Derived Extremophile *Halomonas* sp. MG34

**DOI:** 10.3389/fchem.2020.00197

**Published:** 2020-03-20

**Authors:** Yihong Li, Li Liu, Gengxin Zhang, Ning He, Wenqiang Guo, Bin Hong, Yunying Xie

**Affiliations:** ^1^CAMS Key Laboratory of Synthetic Biology for Drug Innovation, Institute of Medicinal Biotechnology, Chinese Academy of Medical Sciences & Peking Union Medical College, Beijing, China; ^2^Key Laboratory of Alpine Ecology, Institute of Tibetan Plateau Research, China Academy of Sciences, Beijing, China; ^3^NHC Key Laboratory of Biotechnology of Antibiotics, Institute of Medicinal Biotechnology, Chinese Academy of Medical Sciences & Peking Union Medical College, Beijing, China

**Keywords:** potashchelins, lipid siderophore, L-*threo*-β-hydroxyaspartic acid, L-*erythro*-β-hydroxyaspartic acid, *Halomonas*, β-hydroxylases

## Abstract

Four new lipid siderophores bearing both L-*threo*- and L-*erythro*-β-hydroxyaspartic acids, potashchelins A-D (**1**-**4**), were isolated from the potash-salt-ore-derived extremophile *Halomonas* sp. MG34. The planar structures of **1**-**4** were elucidated on the basis of extensive 1D and 2D NMR studies and MS/MS data. Potashchelins **1**-**4** contain a hydrophilic nonapeptide headgroup sequentially consisting of β-hydroxyaspartic acid, serine, glycine, serine, serine, β-hydroxyaspartic acid, threonine, serine, and cyclic N(δ)-hydroxy-ornithine, which is appended by one of a series of fatty acids ranging from dodecanoic acid to tetradecanoic acid. The absolute configurations of the amino acids of potashchelins **1**-**4** were determined by C_3_ and advanced Marfey's reaction, partial hydrolysis, and bioinformatics analysis, which revealed that potashchelins **1**-**4** bear both L-*threo*- and L-*erythro*-β-hydroxyaspartic acid. Phylogenetic analysis showed that the stand-alone β-hydroxylase, PtcA, and the fused domain with β-hydroxylase activity in PtcB are expected to be responsible for the formation of L-*erythro* and L-*threo* diastereomers, respectively. Additionally, utilizing a comparative genomics approach, we revealed an evolutionary mechanism for lipid siderophores in *Halomonas* involving horizontal transfer. Bioassays showed that potashchelin A and D had weak antibacterial activity against *B. subtilis* CPCC 100029 with an MIC value of 64 μg/mL.

## Introduction

Extremophiles are microbes that inhabit extreme environments and represent a fruitful source of natural products (Wilson and Brimble, 2009; Zhang et al., 2018). Extremophiles can be classified as halophiles, alkaliphiles, acidophiles, piezophiles, psychrophiles, thermophiles, etc. based on their optimum growth conditions (Rothschild and Mancinelli, 2001). For example, halophiles thrive in environments with a high salt concentration. In our research, we are dedicated to discovering novel natural products produced by halophiles collected from the Qinghai-Tibet Plateau.

Nonribosomal peptides are assembled by nonribosomal ribosomal peptide synthetases (NRPSs) and play a pivotal role in the treatment of diseases (Sussmuth and Mainz, 2017) and the survival of producer strains (Boiteau et al., 2016). With the deciphering of the biosynthetic logic of nonribosomal peptides and advances in genome sequencing technologies and bioinformatics, many genome mining methods targeting NRPSs have been developed to exploit novel nonribosomal peptides (Chen et al., 2019). Among these methods, PCR-based genome mining using degenerate primers is valued for its ability to rapidly identify strains potentially producing the desired structural class of compounds before the whole genomes of the microbes are sequenced. In recent years, PCR-based genome mining has been successfully utilized to discover novel natural products (Liu et al., 2018; Zhou et al., 2018).

**Figure 1:**
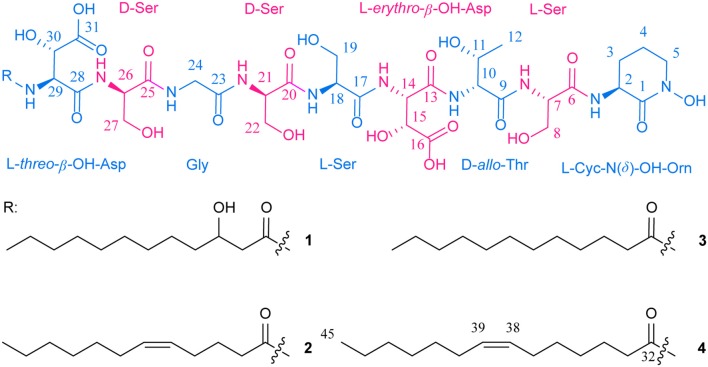
Structures of potashchelins A–D from *Halomonas* sp. MG 34.

Here, we report the discovery of four novel lipid siderophores, potashchelins A-D (**1**-**4**, Figure 1), from *Halomonas* sp. MG34 based on bioassays and PCR-guided screening. The planar structures of **1**-**4** were elucidated by NMR and MS/MS spectral data. The absolute configurations of the amino acids forming these peptides were determined by a combination of Marfey's analysis, partial hydrolysis, and bioinformatics analysis of their biosynthetic gene cluster. Unexpectedly, both L-*threo*- and L-*erythro*-β-hydroxyaspartic acids are present in potashchelins **1**-**4**. Further phylogenetic analysis revealed that the stereospecificity of the β-hydroxylases in the lipid siderophore biosynthesis gene clusters can be predicted, which in turn indicated that the L-*threo* and L-*erythro* diastereomers in potashchelins **1**-**4** are synthesized by the stand-alone β-hydroxylase PtcA and the fused domain with β-hydroxylase activity in PtcB, respectively. Additionally, using a comparative genomics approach, we disclosed an evolutionary mechanism of lipid siderophores in *Halomonas* involving horizontal transfer. Potashchelins A-D (**1**-**4**) were assayed for their antibacterial activity and potashchelin A and D showed weak antibacterial activity against *B. subtilis* CPCC 100029 (64 μg/mL MIC).

## Materials and Methods

### General Experimental Details

UV data were recorded on a Shimadzu UV-2550 spectrophotometer. NMR data were acquired with Varian Mercury 600 spectrometers using DMSO-*d*_6_ as solvent. HRESIMS and ESIMS/MS data were recorded on a Thermal LTQ Orbitrap XT mass spectrometer. HPLC analyses were performed on an Agilent 1200 or Shimadzu DGU-20A instrument using an XBridge C_18_ column (3.5 μm, 4.6 × 150 mm) on a binary LC system [solvent A: 0.1% (v/v) TFA aqueous], solvent B: acetonitrile containing 0.1% (v/v) TFA as modifier; flow rate, 1 mL/min; 0–40 min, 25–45% B (linear gradient); UV detection at 215 nm and oven temperature at 25°C. HPLC purifications were carried out using a XBridge™ Prep C_18_ (5 μm, 10 × 150 mm); eluted with 0.1% (v/v) TFA aqueous (solvent A)−0.1% (v/v) TFA in MeCN (solvent B) from 37% to 45% B during 50 min; flow rate, 2.5 mL/min; UV detection at 215 nm and oven temperature at 25°C. The optimized eluents 37, 40, 42, and 45% acetonitrile aqueous inclusive of 0.1% TFA modifier were used to repurify potashchelins A-D, respectively.

### Isolation, Screening, and Identification of *Halomonas* sp. MG34

Fifteen extremophile strains were isolated from potash salt ore, collected in Qinghai-Tibet Plateau, China and cultured in the medium (S5): 30 g/L NaCl, 100 g/L MgCl_2_, 5 g/L MgSO_4_·7H_2_O, 5 g/L soya peptone, 3 g/L yeast extract, 18 g/L agar, adjusted to pH 7.2, supplemented with 3% NaCl.

For PCR screening of potential producers of nonribosomal peptides, the genomic DNAs from all 15 strains were extracted using standard protocols (Kieser et al., 2000). The degenerate primers of A3F (5′-GCSTACSYSATSTACACSTCSGG-3′) and A7R (5′-SASGTCVCCSGTSCGGTAS-3′) were used in the process of screening (Ayuso-Sacido and Genilloud, 2005). A 50 μL PCR system containing 2 μL of forward primer (10 μM), 2 μL of reverse primer (10 μM), 2 μL genomic DNA, 25 μL of Easy Taq Polymerase (Beijing TransGen Biotech, Beijing, China), and 19 μL of sterilized water, was used. The PCR program was 95°C/5 min [95°C/30 s, 59°C/2 min, 72°C/4 min] × 35 cycles, 72°C/10 min. The PCR products were analyzed by agarose gel electrophoresis and the expected size was 700–800 bp. In all of the 15 halophile strains screened, NRPS sequences were detected (Figure S1).

For bioassay screening of potential producers with antibacterial activity, the spores of each of the 15 strains were inoculated into thirteen different media (see Table S1) and cultured at 28°C, 180 rpm for 5 days. The fermentation broth was centrifugated and the supernatant was tested for their activity against *B. subtilis* CMCC 100027, *M. phlei* CMCC 160023, *S. aureus* ATCC 29213, *E. coli* ATCC 25922, *P. aeruginosa* ATCC 27853, and *C. albicans* ATCC 10231 using a modified cylinder plate method. The cylinders were put on the surface of agar plate with various tested bacteria and the fermentation broth supernatant was added into the cylinders. The antibacterial activity can be detected by the inhibition zone. The strain MG34 was picked out because it exhibited antibacterial activity only in high salt media of DEF-15 (+) containing 3% NaCl (Table S1).

The *Halomonas* sp. MG34 was taxonomically identified based on the housekeeping 16S rRNA gene. Briefly, the housekeeping 16S rRNA gene was amplified by PCR and sequenced using the primers 27F (5′-AGAGTTTGATCCTGGCTCAG-3′) and 1492R (5′-GGTTACCTTGTTACGACTT-3′). Blast on the EzTaxon-e server (http://www.ezbiocloud.net/) disclosed that its 16S rRNA gene sequence (GenBank accession no. MN636765) shows high identity with those of *Halomonas* strains. The phylogenetic trees, based on the 16S rRNA gene sequences of the strain MG34 and the related *Halomonas* homologs, identified MG34 as a *Halomonas* strain (Figure S2).

### Scale-Up Fermentation and Isolation

The spores of *Halomonas* sp. MG34 were inoculated into high salt medium of DEF-15 (+) (Table S1) to be precultured at 28°C and 220 rpm for 48 h. Then, 50 mL of preculture was transferred into 5 L of Erlenmeyer flasks containing 1 L of DEF-15 (+) medium and incubated on a rotary shaker at 220 rpm, and 28°C. After 5 days, the culture broth was harvested. The fermentation broth was filtered to remove the mycelia and 10 L of filtrate was obtained. Then the target compounds were enriched from the filtrate using a column of macroporous absorbent resin 4006 (1 L, 7.2 × 27 cm). After washing with 5 L of water, the active absorbed materials were eluted with 2 L of 20% and 2 L of 50% aqueous acetone, which were combined and lyophilized to afford 500 mg of crude extract. Antibiotic activity was determined by a paper-disk agar diffusion assay against *S. aureus* ATCC 29213 on Mueller-Hinton medium. The crude extract was further purified utilizing semi-preparative reversed phase HPLC chromatography (Figure S3), running with H_2_O/MeCN containing 0.1% TFA, by repeated preparation to afford potashchelins A (**1**, 3 mg), B (**2**, 5 mg), C (**3**, 3 mg), D (**4**, 4 mg).

Potashchelin A (**1**): white powder; ${[\alpha ]}_{\text{D}}^{25}$ +77.8 (c 0.05, MeOH); UV (MeOH) λ_max_ (log ε) 212 (5.13); 1D and 2D NMR (600 MHz, DMSO-*d*_6_) (see Table 1 and Supplementary Material); HRESI(+)MS [M + H]^+^ *m/z* 1097.4900 (calcd for C_43_H_73_N_10_O_23_, 1097.4850).

**Table 1:** ^1^H (600 MHz) and ^13^C (150 MHz) NMR data for potashchelin A (**1**), B (**2**), C (**3**), and D (**4**) in DMSO-*d*_6_.

**Pos**	**Potashchelin A (1)**		**Potashchelin B (2)**		**Potashchelin C (3)**		**Potashchelin D (4)**	
	**δc**	**δ_**H**_ (*J* in Hz)**	**δc**	**δ_**H**_ (*J* in Hz)**	**δc**	**δ_**H**_ (J in Hz)**	**δc**	**δ_**H**_ (*J* in Hz)**
**Cyclic, N-OH Ornithine**								
C1	164.9		164.9		164.9		164.9	
C2	49.8	4.31, m	49.8	4.31, m	49.8	4.33, m	49.8	4.33, m
C3	27.1	1.91, m	27.1	1.86, m	27.1	1.87, m	27.1	1.87, m
		1.69, m		1.69, m		1.69, m		1.69, m
C4	20.3	1.92, m	20.3	1.90, m	20.3	1.89, m	20.3	1.89, m
C5	51.2	3.49, m	51.2	3.49, m	51.2	3.49, m	51.2	3.49, m
		3.46, m		3.46, m		3.46, m		3.46, m
N1		8.11, d (8.9)		8.11, d (8.4)		8.11, d (8.4)		8.11, d (8.4)
**Serine**								
C6	169.7		169.7		169.7		169.7	
C7	54.9	4.37, dd (13.3, 5.6)	54.9	4.36, dd (13.3, 5.6)	54.9	4.36, dd (13.3, 5.6)	54.9	4.36, dd (13.2, 5.6)
C8	61.8	3.59, dd (10.9, 5.5)	61.8	3.59, dd (10.9, 5.5)	61.8	3.59, dd (10.8, 5.4)	61.8	3.58, m
N2		7.97, d (7.7)		7.96, d (7.7)		7.96, d (7.7)		7.96, d (7.6)
**Threonine**								
C9	169.7		169.7		169.7		169.7	
C10	58.5	4.17, dd (7.7,7.0)	58.6	4.17, t (7.2)	58.6	4.17, t (6.6)	58.6	4.17, t (7.3)
C11	66.8	3.83, m	66.8	3.82, m	66.8	3.83, m	66.8	3.83, m
C12	19.7	1.04, d (6.3)	19.7	1.04, d (6.3)	19.7	1.04, d (6.3)	19.7	1.04, d (6.3)
N3		7.62, d (8.1)		7.61, d (8.1)		7.61, d (8.1)		7.61, d (8.1)
**β-OH aspartic acid**								
C13	168.2		168.2		168.2		168.2	
C14	55.7	4.80, dd (8.6, 3.4)	55.7	4.79, dd (8.6, 3.4)	55.7	4.79, dd (8.6, 3.4)	55.7	4.79, dd (8.6, 3.3)
C15	71.0	4.12, d (3.4)	71.0	4.12, d (3.3)	71.0	4.12, d (3.3)	71.0	
C16	172.5		172.5		172.5		172.5	
N4		8.41, d (8.6)		8.41, d (8.6)		8.39, d (8.6)		8.40, d (8.5)
**Serine**								
C17	170.2		170.2		170.2		170.2	
C18	55.4	4.29, m	55.4	4.29, m	55.4	4.30, m	55.4	4.30, m
C19	61.8	3.61, d (5.7)	61.8	3.61, d (5.7)	61.8	3.61, d (5.4)	61.8	3.61, d (5.0)
N5		7.76, d (8.0)		7.76, d (7.7)		7.74, d (7.6)		7.74, d (7.5)
**Serine**								
C20	170.0		170.0		170.0		170.0	
C21	55.3	4.28, m	55.3	4.28, m	55.3	4.28, m	55.3	4.28, m
C22	61.6	3.54, m	61.6	3.54, m	61.5	3.54, m	61.6	3.54, m
N6		7.80, d (7.8)		7.76, d (7.7)		7.76, d (8.2)		7.76, d (8.0)
**Glycine**								
C23	169.3		169.4		169.4		169.4	
C24	42.2	3.79, d (6.0)	42.2	3.79, d (5.7)	42.2	3.79, t (5.6)	42.2	3.79, t (5.6)
N7		8.12, t (5.7)		8.13, t (5.7)		8.13, t (5.7)		8.13, t (5.7)
**Serine**								
C25	170.2		170.2		170.2		170.2	
C26	54.6	4.44, m	54.6	4.44, dd (13.3, 6.5)	54.6	4.44, dd (13.3, 6.5)	54.6	4.44, dd (13.2, 6.5)
C27	61.9	3.54, m	61.9	3.54, m	61.9	3.54, m	61.9	3.54, m
		3.66, dd (10.6, 5.7)		3.66, dd (10.5, 5.6)		3.66, dd (10.5, 5.7)		3.66, m
N8		7.97, d (7.7)		7.96, d (7.7)		7.96, d (7.7)		7.96, d (7.6)
**β-OH aspartic acid**								
C28	168.8		168.8		168.7		168.7	
C29	55.4	4.73, dd (9.0, 2.6)	55.4	4.73, dd (9.2,2.6)	55.3	4.75, dd (9.2, 2.6)	55.2	4.75, dd (9.2, 2.5)
C30	70.1	4.50, d (2.7)	70.1	4.49, d (2.5)	70.4	4.49, d (1.8)	70.4	4.49, br s
C31	173.0		172.9		172.9		172.9	
N9		7.91, d (9.0)		7.88, d (9.2)		7.86, d (9.2)		7.87, d (9.2)
**Fatty acid tail**								
C32	171.1		172.4		172.5		172.5	
C33	43.3	2.31, dd (14.1, 5.1)	34.9	2.16, dd (8.2, 6.5)	35.3	2.15, t (7.5)	35.2	2.15, t (7.4)
		2.23, dd (14.1, 7.0)						
C34	67.4	3.79, m	25.5	1.51, m	25.3	1.46, br s	25.1	1.47, m
C35	36.5	1.35, m	26.3	1.99, m	28.6	1.24, br s	28.3	1.24, m
C36	25.2	1.26, m	129.1	5.34, m	28.9	1.24, br s	29.0	1.26, m
C37	29.1	1.24, m	130.1	5.34, m	28.9	1.24, br s	26.5	1.98, m
C38	29.1	1.24, m	26.6	1.97, m	29.1	1.24, br s	129.6	5.33, m
C39	29.0	1.24, m	29.1	1.24, m	29.0	1.24, br s	129.6	5.33, m
C40	28.7	1.24, m	28.3	1.24, m	28.7	1.24, br s	26.6	1.98, m
C41	31.3	1.24, m	31.2	1.24, m	31.3	1.24, br s	29.1	1.26, m
C42	22.1	1.26, m	22.1	1.26, m	22.1	1.27, m	28.3	1.24, m
C43	14.0	0.86, t (6.9)	14.0	0.86, t (6.9)	14.0	0.85, t (7.0)	31.1	1.24, m
C44	–	–	–	–	–	–	22.1	1.24, m
C45	–	–	–	–	–	–	13.9	0.85, t (6.8)

Potashchelin B (**2**): white powder; ${[\alpha ]}_{\text{D}}^{25}$ 0 (c 0.05, MeOH); UV (MeOH) λ_max_ (log ε) 202 (3.75); 1D and 2D NMR (600 MHz, DMSO-*d*_6_) (see Table 1 and Supplementary Material); HRESI(+)MS [M + H]^+^ *m/z* 1079.46915 (calcd for C_43_H_71_N_10_O_22_, 1079.4744).

Potashchelin C (**3**): white powder; ${[\alpha ]}_{\text{D}}^{25}-$158.4 (c 0.05, MeOH); UV (MeOH) λ_max_ (log ε) 206 (3.42); 1D and 2D NMR (600 MHz, DMSO-*d*_6_) (see Table 1 and Supplementary Material); HRESI(+)MS [M + H]^+^ *m/z* 1081.48462 (calcd for C_43_H_73_N_10_O_22_, 1081.4901).

Potashchelin D (**4**): white powder; ${[\alpha ]}_{\text{D}}^{25}-$7.92 (c 0.05, MeOH); UV (MeOH) λ_max_ (log ε) 206 (3.47); 1D and 2D NMR (600 MHz, DMSO-*d*_6_) (see Table 1 and Supplementary Material); HRESI(+)MS [M + H]^+^ *m/z* 1107.50574 (calcd for C_45_H_75_N_10_O_22_, 1107.5057).

### Marfey's Analyses

#### C_3_ Marfey's Analysis

C_3_ Marfey's analysis was carried out following the reported method (Kreutzer et al., 2012; Vijayasarathy et al., 2016). Briefly, compounds **1**–**4** (50 μg each) were hydrolyzed in 6 M HI (100 μL) at 115°C for 5 h. Then, the hydrolysates were concentrated to dryness at 115°C for 1 h under a stream of dry N_2_. Subsequently, the hydrolysates were treated with 1 M NaHCO_3_ (30 μL), and then with L-FDAA (1% solution in acetone, 40 μL) at 40°C for 12 h, after which the reaction was neutralized with 1 M HCl (30 μL) and diluted with 500 μL 10% acetonitrile in water prior to HPLC-ESIMS analysis. Authentic standards of L-Ser, D-Ser, L-Orn, D-Orn, L-Thr, D-Thr, DL-*threo*-β-OH-Asp, and DL-*allo*-Thr were derivatized with L-FDAA according to the above method. 2 μL of each derivative was analyzed using HPLC-ESIMS on an Agilent Zorbax SB-C_3_ column (5 μm, 150 × 4.6 mm, 50°C, 1 mL/min) with a gradient elution using H_2_O (mobile phase A) and MeCN (mobile phase B) containing formic acid (0.1%). The gradient elution program was 17% B from 0 to 20 min, 17–45% B from 20 to 30 min, 45–17% B from 30 to 32 min, and 17% B from 32 to 40 min. The presence of each amino acid was assessed by UV (340 nm) and MS, and then their retention times were compared with those from the authentic standard derivatives. The measured retention times (t_R_, min) of authentic L-FDAA derivatives were as follows (min): L-FDAA-L-Ser (9.4), L-FDAA-D-Ser (10.6), L-FDAA-L-Orn (3.7), L-FDA-D-Orn (3.3), L-FDAA-L-*threo*-β-OH-Asp (6.0), L-FDAA-D-*threo*-β-OH-Asp (5.5), L-FDAA-L-*allo*-Thr (11.7) and L-FDAA-D-*allo*-Thr (15.4) (Figure S4). L-FDAA derivatized hydrolysates of **1**-**4** gave retention times of (t_R_, min): L-FDAA-L-Ser (9.4), L-FDAA-D-Ser (10.6), L-FDAA-L-Orn (3.7), L-FDAA-L-*threo*-β-OH-Asp (6.0), L-FDAA-D-*allo*-Thr (15.4) and D or L-*erythro*-β-OH-Asp (10.1) (Figure S4).

#### Advanced Marfey's Method

To determine the stereochemistry at C-2 in *erythro*-β-OH-Asp residues in potashchelins, advanced Marfey's method was used. Briefly, **1**-**4** (50 μg each) were hydrolyzed completely and derivatized with L- and D- FDLA using the same method as above. Authentic standard DL-*threo*-β-OH-Asp was derivatized with L-FDLA according to the above method. An aliquot (2 μL) of each derivative was analyzed using HPLC-ESIMS on an Agilent Zorbax SB-C_3_ column (5 μm, 150 × 4.6 mm, 50°C, 1 mL/min) with a gradient elution using H_2_O (mobile phase A) and MeCN (mobile phase B) containing formic acid (0.1%). The gradient elution program was 27% B from 0 to 20 min, 27–45% B from 20 to 30 min, 45–27% B from 30 to 32 min and 27% B from 32 to 40 min. Authentic L-FDLA derivatized DL-*threo*-β-OH-Asp gave retention times (t_R_, min): L-FDLA-D-*threo*-β-OH-Asp (7.2), and L-FDLA-L-*threo*-β-OH-Asp (7.3). L-FDLA derivatized hydrolysates of **1**-**4** gave retention times of (t_R_, min): D-FDLA-L-*threo*-β-OH-Asp (7.2), L-FDLA-L-*threo*-β-OH-Asp (7.3), D-FDLA-L-*erythro*-β-OH-Asp (10.0), and L-FDLA-L*-erythro*-β-OH-Asp (10.5). Therefore, the *erythro*-β-OH-Asp residues in potashchelins were all determined as L configuration.

#### Partial Hydrolysis

To determine the position of L-*erythro*- and L-*threo*-β-OH-Asp residues in potashchelins, **2** (300 μg) was partially hydrolyzed in 0.5 M HCl (100 μL) at 100°C for 40 min and then the hydrolysates were concentrated to dryness at 100°C for 40 min under a stream of dry N_2_. The hydrolysates were treated with 1 M NaHCO_3_ (30 μL), diluted with 500 μL 10% acetonitrile in water, and then subjected to HPLC (Agilent Zorbax SB-C_3_ column, 5 μm, 150 × 4.6 mm, 30°C, 1 mL/min) with a gradient elution using H_2_O (mobile phase A) and MeCN (mobile phase B) containing formic acid (0.1%). The gradient elution program was 35% B from 0 to 10 min, 35–95% B from 10 to 11 min, 95% B from 11 to 15 min, 95–35% B from 15 to 16 min and 35% B from 16 to 20 min. The main hydrolysis product giving an ion at *m/z* 496 corresponding to fatty-acid-L-β-OH-Asp-Ser-Gly was picked out and purified (Figure S5). Then, it was treated and analyzed using the above mentioned advanced Marfey's method. The retention time of the L-FDLA derivatized L-β-OH-Asp based on SIE (m/z 466 [M+Na]^+^) was 7.5 min (*m/z* 466), which is identical to that of L-FDLA derivatized L-*threo*-β-OH-Asp.

### Genomic DNA Sequencing, Assembly and Bioinformatics Analysis

The strain *Halomonas* sp. MG34 was grown in tryptic soy broth liquid medium and genomic DNA was extracted using a standard chloroform protocol (Nikodinovic et al., 2003). The genome was sequenced using a next generation sequencing platform in the paired-end (2 × 300) format, resulting in 824 Mb clean data (2,764,928 reads with 298 bp average insert size and 110-fold average coverage). The genome was assembled with the SPAdes algorithm (V3.13.1) (Bankevich et al., 2012) to yield the first version of the draft genome. Then, to obtain the complete biosynthetic gene cluster (BGC) of potashchelins, antiSMASH and end-extending method (Huang et al., 2013) were employed. The genome was submitted to the National Center for Biotechnology Information (NCBI) database with the accession No. WJPH00000000. The biosynthetic gene cluster of potashchelins and its domain were identified in the assembled genome using standalone antiSMASH 5. The homologs of PtcA were collected from MIBiG (https://mibig.secondarymetabolites.org/, as of October, 2019). The maximum-likelihood phylogenetic tree of PtcA and its homologs were reconstructed in PhyML 3.2.0, using the LG amino acid substitution model (Guindon et al., 2010).

### Antibacterial Bioassay Method

The MICs for *B. subtilis* CMCC 100027, *M. phlei* CMCC 160023, *S. aureus* ATCC 29213, *E. coli* ATCC 25922, *P. aeruginosa* ATCC 27853, and *C. albicans* ATCC 10231 were determined by a microdilution method (Cockerill et al., 2012). Briefly, the bacterial strain was grown on Mueller-Hinton broth (MHB), and the final suspension of bacteria (in MHB medium) was 10^6^ cells/mL. The fungus, *C. albicans* ATCC 10231, was cultured on SDB, and the final suspension concentration was the same as the bacteria. Tested samples were dissolved in DMSO and diluted serially. Then 1 μL of each diluted sample was added into a 96-well plate in triplicate containing 100 μL of the bacterial suspension in each well. After incubation at 37°C for 18 h, the growth of the tested organism was detected by eye, and the MIC was identified as the lowest concentration that completely inhibited growth of the organism. The positive controls were as the following (MIC, μg/mL): streptomycin for *B. subtilis* (8), *M. phlei* (16) and *M. smegmatis* (16), gentamycin for *E. coli* (2) and *P. aeruginosa* (2), vancomycin for *S. aureus* (2), amphotericin B for *C. albicans* (8). The MIC values of potashchelins A–D are shown in Table 2.

**Table 2:** Antimicrobial bioassay results (MIC, μg/mL) for potashchelins A–D (**1**–**4**).

**Compounds**	***B. subtilis CPCC 100029***	***M. phlei*CPCC 160023**	***M. smegmatis***	***E. coli*ATCC 25922**	***P. aeruginosa* 11**	***S. aureus*ATCC 29213**	***C. albicans* ATCC 10231**
Potashchelin A (**1**)	64	>64	>64	>64	>64	>64	>64
Potashchelin B (**2**)	>64	>64	>64	>64	>64	>64	>64
Potashchelin C (**3**)	>64	>64	>64	>64	>64	>64	>64
Potashchelin D (**4**)	64	>64	>64	>64	>64	>64	>64
Streptomycin	8	16	16	*ND*	*ND*	*ND*	*ND*
Gentamycin	*ND*	*ND*	*ND*	2	2	*ND*	*ND*
Vancomycin	*ND*	*ND*	*ND*	*ND*	*ND*	2	*ND*
Amphotericin B	*ND*	*ND*	*ND*	*ND*	*ND*	*ND*	8

*ND, Not detected*.

## Results

### Strain Prioritization for Nonribosomal Peptide Discovery From Halophiles Based on the Combination of PCR and Bioassay Screening

The combination of PCR and bioassay screening was used to discover active nonribosomal peptides from halophiles isolated from samples collected from the Qinghai-Tibet Plateau. The degenerate primers A3F and A7R, deduced from the conserved sequences of the adenylation domains (A) of NRPSs, were designed to amplify NRPS gene sequences from Actinomycetes and proved to be very useful for strain prioritization (Ayuso-Sacido and Genilloud, 2005; Lemetre et al., 2017; Masand et al., 2018; Zhou et al., 2018). First, 15 halophile strains were screened using PCR primers A3F and A7R. To our surprise, NRPS sequences were detected in all 15 halophile strains screened by PCR (Figure S1). The positive rate of 100% in the PCR assay indicates that all of the tested strains have the potential to produce nonribosomal peptides. Then, each of the 15 strains was fermented on a small scale (100 mL) using 13 different types of media and prioritized by antibacterial activity. Strain MG34 was selected because it exhibited antibacterial activity only in DEF-15 (+) high-salt medium, which contained 3% NaCl (Table S1). To identify the genus of strain MG34, the housekeeping 16S rRNA gene was amplified by PCR and sequenced. Blast on the EzTaxon-e server (http://www.ezbiocloud.net/) revealed that the 16S rRNA gene sequence of MG34 showed high identity with those of *Halomonas* strains. Phylogenetic trees based on the 16S rRNA gene sequences of MG34 and related *Halomonas* homologs identified MG34 as a *Halomonas* strain (Figure S2).

### Production and Isolation of Potashchelins A-D

*Halomonas* sp. MG34 was fermented in DEF-15 (+) high-salt medium, and potashchelins A-D were isolated and purified using a bioassay-guided method from a 5-day broth culture. Briefly, the fermentation broth of *Halomonas* sp. MG34 was filtered, and potashchelins were enriched from the filtrate by a macroporous absorbent resin 4006 column. The yielded active crude extract was analyzed by HPLC, but no apparent peaks appeared when an aqueous solution of MeOH or MeCN was used as an eluent. When TFA was added to the eluent as a modifier, five peaks appeared on the HPLC spectrum with excellent resolution (Figure S3). Therefore, the target peaks were further purified utilizing semi-preparative reversed phase HPLC chromatography (run with H_2_O/MeCN containing 0.1% TFA) to yield potashchelins A (**1**, 3 mg), B (**2**, 5 mg), C (**3**, 3 mg), and D (**4**, 4 mg).

### Planar Structural Elucidation by Spectroscopic Analysis

Potashchelin A (**1**) was obtained as a white powder and proved to have a molecular formula of C_43_H_73_N_10_O_23_, based on high resolution electrospray ionization mass spectrometry (HR-ESIMS) [M + H]^+^ *m/z* 1097.4900 (calcd for C_43_H_73_N_10_O_23_, 1097.4850). Analysis of the ^1^H and ^13^C NMR data for **1** (Table 1) revealed the presence of twelve ester/amide carbonyls (δ_C_ 164.9–172.4), accounting for twelve double bond equivalents and requiring that **1** incorporate one ring. Further interpretation of ^1^H-^1^H COSY, HSQC, and HMBC spectroscopic data (Supplementary Material) disclosed correlations, indicative of a nonapeptide consisting of a cyclic N(δ)-hydroxyornithine (C1-C5), four serines (C6-C8, C17-C19, C20-C22, and C25-C27), two β-hydroxyaspartic acid (C13-C16 and C28-C31), one threonine (C9-C12), and one glycine (C23 and C24), as well as a fatty acid tail (C32-C43). The presence of these amino acid residues was also confirmed by C_3_ Marfey analysis (see below). The fatty acid tail was evidenced to be 3-hydroxydodecanoic acid by comprehensive analysis of 1D and 2D NMR data, as well as comparison with the reported data in the literature (Frost and Gunstone, 1975; Gunstone et al., 1977).

**Figure 2:**
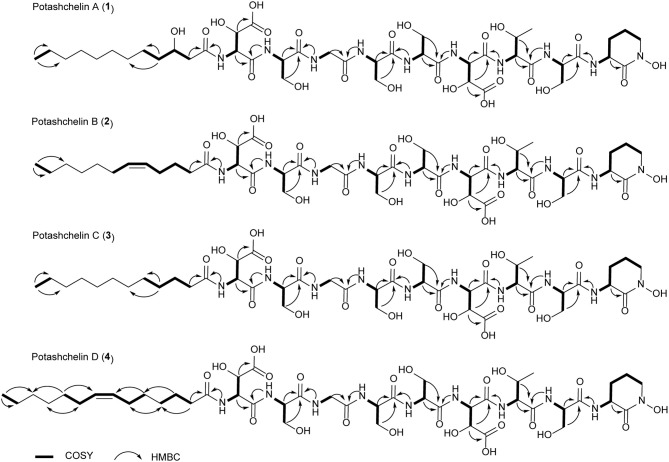
^1^H-^1^H COSY and selected HMBC correlations for potashchelins A–D.

**Figure 3:**
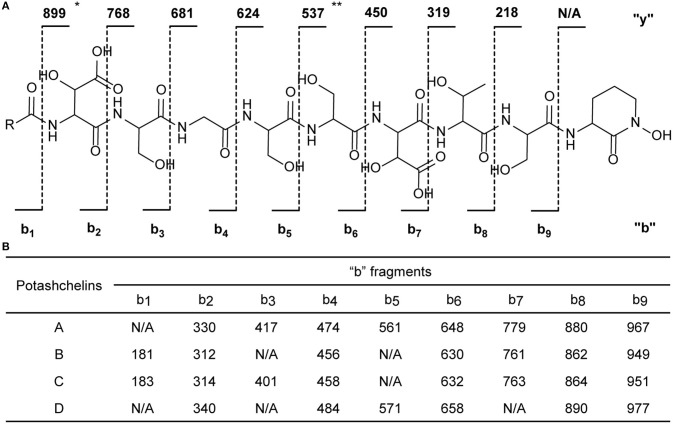
Tandem mass spectrometry fragmentation analysis of potashchelins A–D. **(A)** The fragmentation patterns of potashchelins. R represents the different fatty acid tails of potashchelins A–D. “y” and “b” represent the “y” and “b” fragments, respectively. The “y” fragment *m/z* values are the same for potashchelins A–D. **(B)** The “b” fragment *m/z* values for potashchelins A–D. ^*^: Not applicable for potashchelin A. ^**^: Not applicable for potashchelins B and C.

The two-bond HMBC correlations (Figure 2) from amide NH protons of cyclic N(δ)-hydroxyornithine (δ_H_ 8.11), serine (δ_H_ 7.97), threonine (δ_H_ 7.62), β-hydroxyaspartic acid (δ_H_ 8.41), serine (δ_H_ 7.76), serine (δ_H_ 7.80), glycine (δ_H_ 8.12), serine (δ_H_ 7.97), and β-hydroxyaspartic acid (δ_H_ 7.91) to the carbonyl carbons of serine (δc 169.7), threonine (δc 169.7), β-hydroxyaspartic acid (δc 168.2), serine (δc 170.2), serine (δc 170.0), glycine (δc 169.3), serine (δc 170.2), β-hydroxyaspartic acid (δc 168.8), and the fatty acid tail (δc 171.1), respectively, preliminarily identified the sequence of nine amino acids and the fatty acid as fatty-acid-β-OH-Asp-Ser-Gly-Ser-Ser-β-OH-Asp-Thr-Ser-cyclic-N(δ)-OH-Orn. This sequence was further confirmed by the tandem mass spectrometry fragmentation pattern (Figure 3). The “y” fragments *m/z* 218, 319, 450, 537, 624, 681, and 768, were attributed to the sequential increase of 218, 101, 131, 87, 87, 57, and 87, corresponding to the amino acids cyclic-N(δ)-OH-Orn-Ser, Thr, β-OH-Asp, Ser, Ser, Gly, and Ser, from the C-terminus. On the other hand, the “b” fragments *m/z* 967, 880, 779, 648, 561, 474, 417, and 330, owing to sequential loss of cyclic-N(δ)-OH-Orn, Ser, Thr, β-OH-Asp, Ser, Ser, Gly, and Ser, also confirmed the above sequence.

High-resolution ESIMS of potashchelin B (**2**) yielded *m/z* 1079.46915 for [M + H]^+^ quasi-molecular ion, indicative of a molecular formula of C_43_H_71_N_10_O_22_, a shortage of an “H_2_O” compared with that of **1**. Tandem mass spectrometry exhibited that each corresponding “b” fragment in **2** was decreased by 18 Da (Figure 3) compared with that of **1**, suggesting that **2** differ from **1** in their fatty acid tail, which was further confirmed by the NMR data. The absence of the carbon signal at δ 67.4 in ^13^C NMR spectrum of **2**, compared with that of **1**, indicated the loss of the hydroxy group at C34. In addition, compared with the ^13^C NMR spectrum of **1**, that of **2** showed two additional *sp*^2^ carbon signals at δ 129.1 and 130.1, while two disappeared *sp*^3^ carbon signals at δ 25.2 and 29.1, indicating the presence of a double bond in the fatty acid tail of **2**, the position of which was further determined to be between C36 and C37 by the 1D and 2D NMR. Then we tried to elucidate the configuration of the double bond using the *J* value of olefinic protons, but unfortunately, the complicated absorption produced by the two olefinic protons (-CH=CH-) with quite close chemical shifts made it impossible to calculate the coupling constants accurately. However, the *cis* configuration of the double bond can be undoubtedly determined by the δ values below 30 ppm (26.3 and 26.6) of the allylic carbons adjacent to the double bond (Gunstone et al., 1977). Thus, the fatty acid in **2** was identified as (*Z*)-dodec-5-enoic acid.

Potashchelin C (**3**) has a molecular formula of C_43_H_73_N_10_O_22_, based on high resolution electrospray ionization mass spectrometry (HR-ESIMS) [M + H]^+^ *m/z* 1081.48462, 16 mass units smaller than that of **1**. The tandem mass spectrometry showed similar fragmentation patterns with those of **1**, except that each “b” fragment of **3** was decreased by 16 Da (Figure 3), indicative of the loss of a hydroxyl group in the fatty acid tail of **3**. The absence of carbon and proton signals [δ_C_ 67.4 and δ_H_ 3.79 (1H, m)] produced by -CH2-OH in the NMR spectra of **3** confirmed the proposed structure.

High-resolution ESIMS of potashchelin D (**4**) gave the quasi-molecular ion [M + H]^+^ *m/z* 1107.50574, suggestive of the molecular formula of C_45_H_75_N_10_O_22_, which possessed an extra C_2_H_2_ in comparison to that of **2**. Compound **4** also had the similar “y” MS/MS fragments as **1**-**3**, while different “b” fragments, each of which was 24 mass units greater than those of **2**, hinting that the extra C_2_H_2_ was located in the fatty acid tail. These results suggested that **4** could have a tetradecenoic acid tail. This hypothesis was further identified by the ^1^H and ^13^C NMR spectra of **4**, which exhibited extra signals [δ_C_ 28.3, 29.0 and δ_H_ 1.24 (2H, m), 1.26 (2H, m)]. The extra signals were assigned as CH_2_-35 (δ_C_ 28.3, δ_H_ 1.24) and CH_2_-36 (δ_C_ 29.0, δ_H_ 1.26), based on the ^1^H-^1^H COSY connections between δ_H−34_ 1.47 and δ_H−35_ 1.24, as well as the HMBC connections from δ_H−33_ 2.15 to δ_C−35_ 28.3, and δ_H−34_ 1.47 to δ_C−36_ 29.0 (Figure 2). Furthermore, the ^1^H-^1^H COSY spectrum disclosed that H-36 (δ_H_ 1.26) correlated to allylic methylene protons (δ_H_ 1.98), which in turn connected with the olefinic proton (δ_H−38_ 5.33), indicative of the position of the double bond between C-38 and C-39. TheΔ7 position and *cis* configuration of the double bond in the fatty acid of **4** were further confirmed by comprehensive interpretation of 1D and 2D NMR data of **4** (Table 1 and Figure 3) and comparison with those of **2**. Therefore, **4** contained (*Z*)-tetradec-7-enoic acid.

### Absolute Configuration Determination by the Combination of Marfey's Method, Partial Hydrolysis and Bioinformatics Analysis

**Figure 4:**
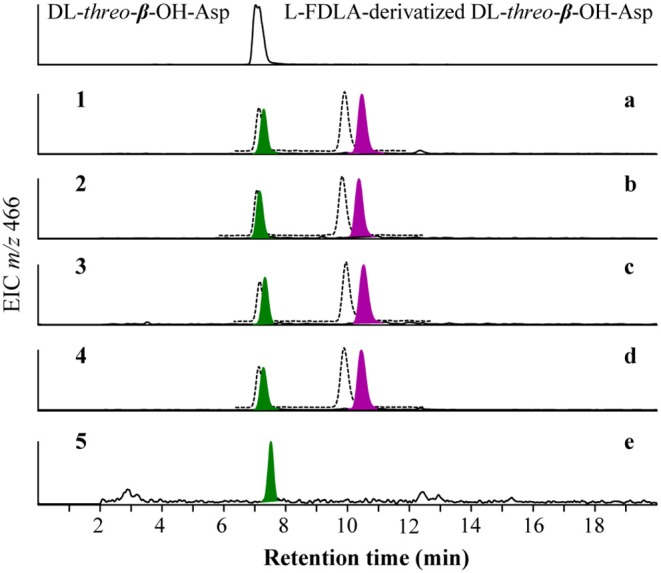
Advanced Marfey's analysis for β-OH-Asp in potashchelins A-D (a–d) and the fragment of fatty-acid-β-OH-Asp-Ser-Gly obtained from the partial hydrolysates of potashchelin B (e). L-FDLA and D-FDLA derivatized β-OH-Asp are indicated in colored and broken-line peaks, respectively.

To resolve the absolute configurations of the amino acid residues, we first applied C_3_ Marfey's method (Vijayasarathy et al., 2016) based on acidic hydrolysis of potashchelins. Reductive HI cleavage was carried out to release two β-hydroxyaspartic acid moieties and an ornithine residue (Kreutzer et al., 2012). After derivatization with Marfey's reagent (L-FDAA), the hydrolysates of **1**–**4** were analyzed by HPLC-DAD-MS and compared with authentic amino acid standards. The analyses revealed the presence of L-Orn, two L-Ser, two D-Ser, D-*allo*-Thr, L-*threo*-β-OH-Asp, and D- or L-*erythro*-β-OH-Asp residues in **1**–**4** (Figure S4). The advanced Marfey's method, in which the hydrolysates of **1**–**4** were derivatized with L-FDLA or D-FDLA, was employed because the standard of *erythro*-β-OH-Asp was unavailable (Fujii et al., 1997a,b). According to the elution order of the diastereomeric pairs of L- and D-FDLA-derivatized *erythro*-β-OH-Asp (Fujii et al., 1997b), the *erythro*-β-OH-Asp residues in **1**–**4** were all determined to exist in the L configuration (Figure 4). Partial hydrolysis was performed to address the regiochemistry of the enantiomeric and epimeric amino acid residues in **1**–**4**. The main fragment, determined to be fatty-acid-β-OH-Asp-Ser-Gly based on mass analysis (Figure S5), was purified, hydrolyzed, derivatized with L-FDLA, and subjected to C_3_ Marfey's analysis. The regiochemistry of the L-*threo*-β-OH-Asp adjacent to the fatty acid tail was unambiguously established (Figure 4), but, unfortunately, that of D-Ser and L-Ser were not determined despite significant effort because only trace amounts of the corresponding partial hydrolysates were present.

Bioinformatics analyses were used to completely clarify the regiochemistry of the enantiomeric serine residues in **1**–**4**. A draft genome sequence of *Halomonas* sp. MG 34 was obtained using next generation sequencing platform and evaluated using antiSMASH 5.0.0 for the presence of a biosynthetic gene cluster (BGC) identical to the NRPS-derived backbone of **1**–**4**. The analysis showed that the putative potashchelin BGC was divided into four subclusters, which were arranged according to the structures of **1**–**4** and the substrate specificities of the adenylation domains of NRPSs. The end-extending method (Huang et al., 2013) was employed to fill the gaps between contigs. All three gaps between these four contigs were filled, and a connected contig containing the complete potashchelin BGC was obtained, which made it possible to determine the regiochemistry of the enantiomeric serine residues in **1**–**4**.

**Figure 5:**
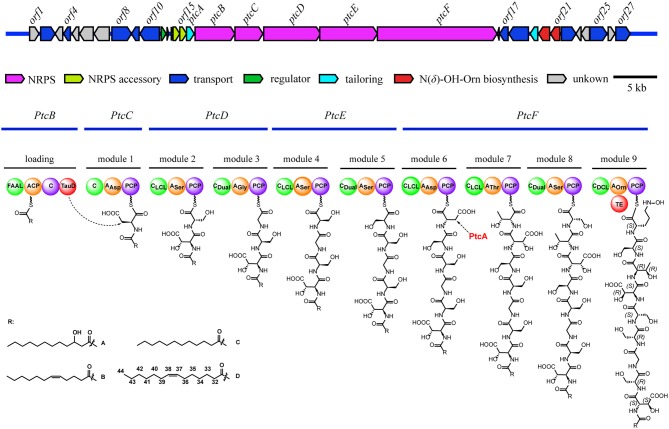
Organization of the potashchelin biosynthetic gene cluster (top) and the proposed biosynthetic pathway for potashchelin assembly (bottom). FAAL, fatty acyl-AMP ligase; C, condensation. The subscript of the C domain indicates its stereospecificity; C_Dual_, condensation domain with epimerization activity; A, adenylation. The substrate of each A domain is indicated as a subscript; PCP, peptidyl carrier protein; TE, thioesterase; TauD, nonheme Fe(II)/α-ketoglutarate dependent dioxygenase.

The architecture and features of the potashchelin BGC were interrogated, and a model for potashchelin biosynthesis was therefore deduced (Figure 5). Based on the antiSMASH and BLAST analyses, ten genes within the BGC (*orf2, 4, 8, 9, 10, 17, 18, 22, 25*, and *27*) were determined to be involved in siderophore transport, while one gene (*orf11*) was associated with regulation of this cluster. *orf20* and *orf21* encode a lysine/ornithine N-monooxygenase and an acyltransferase, respectively, which are proposed to act concertedly to supply the amino acid precursor N(δ)-OH-Orn (Kreutzer et al., 2012), whereas a lipase encoded by *orf19* may be responsible for providing the fatty acid starter unit. In addition, an MbtH-like protein (Zhang et al., 2010), type-II thioesterase (Schwarzer et al., 2002), and 4′-phosphopantethenyl transferase (Flugel et al., 2000) encoded by *orf13, orf14* and *orf15*, respectively, are expected to activate and improve the functionality of the NPRS biosynthesis machinery. The five NRPSs derived from the successive genes *ptcB, ptcC, ptcD, ptcE*, and *ptcF* are responsible for the assembly of the nonapeptide potashchelin backbone. The resulting proteins form ten modules: one for initiation and nine for extension and termination of the NRPS assembly line. The loading module, which has the same domain arrangement as the taiwachelin BGC (Kreutzer and Nett, 2012), primes the fatty acid tail onto the ACP domain and initiates biosynthesis. Next, the nine amino acids constituting a potashchelin are assembled consecutively by the nine extension modules harbored by PtcC, PtcD, PtcE, and PtcF. The last extension module has a TE domain, which may be responsible for cyclization of N(δ)-OH-Orn and offloading the final product.

The adenylation (A) domains, from the specificity-conferring code of which the building blocks of peptides can be predicted, and the condensation (C) domains, from which the configuration of the peptides in these modules can be deduced, are of particular interest, as they account for the selection and stereochemistry of the monomer amino acids incorporated in potashchelins. The sequence of amino acid residues deduced by the biosynthesis analysis was the same as that determined by NMR and tandem MS interpretation. There are no *E* domains in the BGC, but the C_Dual_ domains (Balibar et al., 2005) within the BGC function both as an epimerase and in condensation, so the configurations of amino acids inserted in the peptide backbone were determined by these C domains (Figures S31, S32). Based on the arrangement of the domains in modules 1-9, FAAL-ACP-C-TauD-C-A_Asp_-PCP-C_LCL_-A_ser_-PCP-C_Dual_-A_Gly_-PCP-C_LCL_-A_Ser_-PCP-C_Dual_-A_Ser_-PCP-C_LCL_-A_Asp_-PCP-C_LCL_-A_Thr_-PCP-C_Dual_-A_Ser_-PCP-C_DCL_-A_Orn_-PCP-TE, the complete siderophore should feature L-β-OH-Asp-D-Ser-Gly-D-Ser-L-Ser-L-β-OH-Asp-D-*allo*-Thr-L-Ser-L-Cyc-N(δ)-OH-Orn as the peptide backbone. The presence of two L-Ser and two D-Ser amino acid residues is consistent with the results of Marfey's analysis.

**Figure 6:**
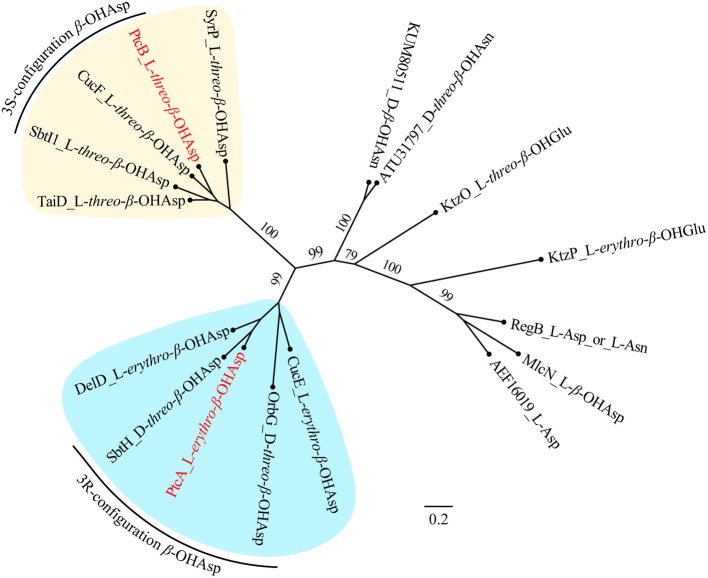
Maximum-likelihood phylogenetic tree of β-hydroxylase PtcA and its homologs collected from MIBiG. The product of each β-hydroxylase is indicated after the name or accession of each enzyme. Two subclades containing enzymes yielding the 3S and 3R configuration of β-OH-Asp, respectively, are highlighted. PtcA and PtcB are paraphyletic with the 3R and 3S configuration reactivity enzymes, respectively. Bootstrap values based on 1,000 resampled datasets are shown on the branches.

NMR and Marfey's methods revealed the presence of L-*threo*-β-OH-Asp and L-*erythro*-β-OH-Asp at the first and sixth amino acid residues, respectively, of **1-4**. PtcA, which has TauD activity, and the TauD domain at the C-terminus of PtcB are expected to be responsible for hydroxylation at the beta carbon of aspartic acid after it bonds to ACP, as in cupriachelin biosynthesis (Kreutzer et al., 2012). It was reported that cupriachelin contains two L-*threo*-β-OH-Asp residues (Kreutzer et al., 2012), but a more recent study identified one L-*threo*-OH-Asp residue and one L-*erythro*-β-OH-Asp residue (Reitz et al., 2019). To assess the stereospecificity of PtcA and the TauD domain at the C-terminus of PtcB, MiBIG-provided homologs of PtcA involved in the beta-hydroxylation of Asp, Glu and Asn were aligned (as of November, 2019). Interestingly, all stand-alone enzymes and fused domains catalyzing the beta carbon of Asp to produce the *R* configuration were clustered together, while those producing the *S* configuration were clustered separately (Figure 6). When PtcA and the PtcB domain with TauD activity were considered together, the latter was clustered with the *S* configuration subclade, while the former was located in the *R* configuration subclade. Therefore, the TauD domain in PtcB can be envisioned to catalyze ACP-Asp in its adjacent module to form L-*threo*-β-OH-Asp, as in taiwachelin biosynthesis (Kreutzer and Nett, 2012), while PtcA is proposed to be involved in the production of L-*erythro*-β-OH-Asp. As this manuscript was nearing completion, a similar analysis about stereospecificity of β-hydroxylases was reported by Reitz et al. (2019), in which a method to predict β-hydroxylase stereochemistry *in silico* was developed through mapping stereochemically characterized β-OH-Asp residues in siderophores to the phylogenetic tree of β-hydroxylases. Our result confirms that the stereospecific reactivity of β-hydroxylases in siderophore biosynthesis can be predicted by aligning their amino acid sequences.

### Evolution of the Potashchelin Gene Cluster

**Figure 7:**
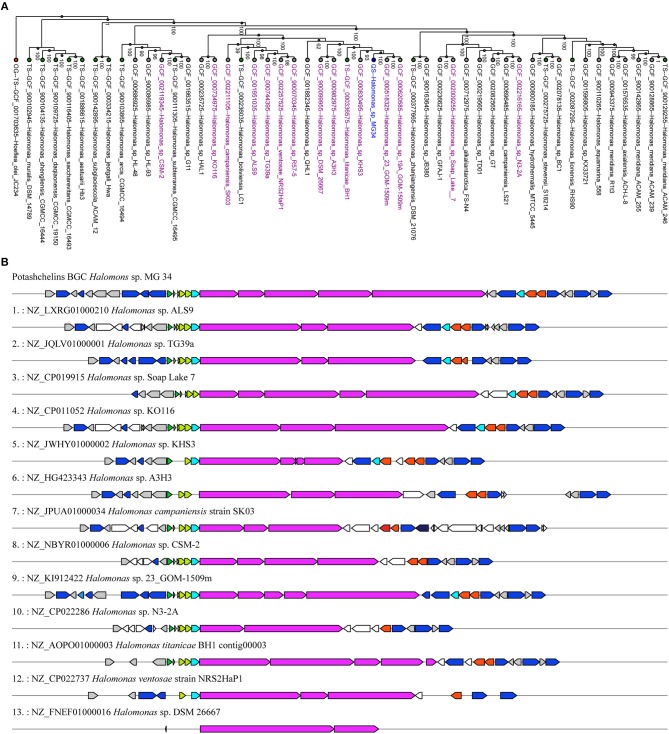
Evolution of lipid siderophore BGCs in *Halomonas* species. **(A)** Phylogeny of multi-locus species tree (MLST) genes (Alanjary et al., 2019) from *Halomonas* sp. MG 34 and its nearest reference organisms (NCBI RefSeq) with NRPS-containing strains indicated in purple. The green and red nodes represent the type strains and outgroup, respectively. *Halomonas* sp. MG 34 is highlighted in blue. The phylogenetic tree was reconstructed using genomic DNA of *Halomonas* sp. MG 34 as a query for http://automlst.ziemertlab.com/ (Alanjary et al., 2019). **(B)** Homologs of the potashchelin BGC in *Halomonas* genome sequences containing the NRP biosynthetic gene cluster indicated by the autoMLST analysis.

Siderophores play a particularly important role in the bioavailability of iron in iron-scarce areas (Boiteau et al., 2016). Lipid siderophores biosynthesized by NRPSs have been found in several *Halomonas* species (Martinez et al., 2000; Homann et al., 2009; Figueroa et al., 2015), which often inhabit iron-poor environments. However, it remains unknown whether *Halomonas* sp. acquired the biosynthetic gene clusters of lipid siderophores by vertical or horizontal gene transfer. To explore this question, we first reconstructed a phylogenetic tree (Figure 7A) using the genomic DNA of *Halomonas* sp. MG34 as a query for the autoMLST server (http://automlst.ziemertlab.com/) (Alanjary et al., 2019), which can quickly provide related strains and annotate their secondary metabolite types. NRPSs were detected in only a few *Halomonas* species (Figure 7A), suggesting that lipid siderophore BGCs were likely acquired by *Halomonas* species via horizontal gene transfer. Then, to better understand the distribution of the potashchelin BGC in *Halomonas*, which is, to the best of our knowledge, the first reported lipid siderophore BGC from this genus, we used this BGC as a multigene BLAST query against all *Halomonas* genome sequences containing a NRP biosynthetic gene cluster indicated by autoMLST analysis (Figure 7B). The potashchelin biosynthesis gene cluster was not detected in any of the tested genomes, suggesting that it is relatively rare in *Halomonas* species. However, this analysis revealed some homologs of potashchelin BGC with highly conserved boundary regions, which might help to define the edges of the potashchelin BGC.

### Antibacterial Activities of Potashchelins

The antibacterial activity of potashchelins A–D (**1**-**4**) was assayed by the micro broth dilution method. Streptomycin, gentamycin, vancomycin, and amphotericin B were selected as positive control treatments. As shown in Table 2, only potashchelins A (**1**) and D (**4**) exhibited weak antibacterial activity against *B. subtilis* CPCC 100029 (MIC = 64 μg/mL), but they had no activity against *E. coli, P. aeruginosa, M. phlei, M. smegmatis, S. aureus*, or *C. albicans* (MIC > 64 μg/mL).

## Discussion

PCR screening based on the conserved sequences of adenylation domain (A) in nonribosomal peptides (NRPs) has been successfully used for the discovery of NRPs. Degenerate primers A3F and A7R were designed to amplify NRPS gene sequences from actinomycetes and proved to be very useful for strain prioritization of actinomyces, with positive rates ranging from 0% to 100% (Ayuso-Sacido and Genilloud, 2005; Lemetre et al., 2017; Masand et al., 2018; Zhou et al., 2018). In this study, using this degenerate primer pair, we successfully amplified NRPS genes from extremophiles and prioritized extremophile strains. The high positive rate of 100% demonstrated the efficacy of the degenerate primers in the amplification of NRPS genes from extremophiles and revealed the richness of the NPSs in the extremophiles tested in this study.

Bioassay-guided prioritization and isolation are traditional methods for the discovery of new compounds with activity. It showed efficiency during the prioritization of tested strains in this study. However, bioassay-guided isolation did not seem to be successful, because the bioactivity of potashchlins A-D was not in line with that detected in the crude extract; the latter showed activity against *S. aureus*, while none of the former compounds exhibited activity against *S. aureus* at the concentration of less than 64 μg/mL. We speculate that this divergence of bioactivity may be aroused by the weak activity of the metabolites against *S. aureus*, or the minor components in the crude extract were not detected, which is often encountered during the process of bioassay-guided isolation.

Lipid siderophores are often secreted by microbes to obtain iron from iron-scarce environments, and these molecules play an important role in the recycling of iron in the environment (Boiteau et al., 2016). β-OH-Asp is often found in lipid siderophores and takes part in covalent bonding with iron ions (Hardy and Butler, 2018). β-OH-Asp bears two stereocenters at C2 and C3, and it exists as four diastereomers: L-*threo*-, D-*threo*-, L-*erythro*-, and D-*erythro*-β-OH-Asp. Most lipid siderophores contain one β-OH-Asp with either L-*erythro*, L-*threo*, or D-*threo* stereochemistry (Stephan et al., 1993; Agnoli et al., 2006; Kreutzer and Nett, 2012; Johnston et al., 2013; Reitz et al., 2019). Only serobactin (Kreutzer et al., 2012; Rosconi et al., 2013) and pacifibactin (Hardy and Butler, 2019) were reported to hold two β-OH-Asp with LD-*threo* diastereomers, while cupriachelin has been reported to bear L-*threo*- and L-*erythro*-β-OH-Asp residues (Kreutzer et al., 2012; Reitz et al., 2019). However, the locations of the β-OH-Asp diastereomers of serobactin, pacifibactin, and cupriachelin have not been chemically determined. Here, we report that potashchelins contain both L-*threo*- and L-*erythro*-β-OH-Asp, and we report the locations of these residues for the first time based on the results of partial hydrolysis and Marfey's analysis.

To determine the configuration of the four serine residues of the potashchelins, the potashchelin biosynthesis gene cluster was deduced by bioinformatics analysis. To the best of our knowledge, this is the first reported lipid siderophore BGC from the genus *Halomonas*. Five NRPSs, encoded by the consecutive genes *ptcB, ptcC, ptcD, ptcE*, and *ptcF*, containing one loading and nine extending modules, are expected to be responsible for assembly of the potashchelin backbone. Importantly, we were able to deduce the stereochemistry of Ser based on our analysis of the C domains in modules 3, 5, 6, and 9 (Figure 1). Additionally, we further explored the evolutionary history of lipid siderophore BGCs using the genomic sequence of *Halomonas* sp. MG34 and the potashchelin BGC as a query for the autoMLST server and Multigeneblast analysis. This analysis suggested that the evolutionary mechanism of lipid siderophores in *Halomonas* involved horizontal transfer, despite the presence of lipid siderophores in several *Halomonas* species (Martinez et al., 2000; Homann et al., 2009; Figueroa et al., 2015).

Two β-hydroxylases (one stand-alone PtcA and one fused with the C-domain in PtcB) belonging to the TauD/TdfA family of nonheme Fe(II)/α-ketoglutarate dependent dioxygenases (Singh et al., 2008) were discovered in the potashchelin BGC and proposed to be involved in hydroxylation of Asp at C-3. When homologs of the potashchelin BGC are considered together, it is interesting that the stereospecificity of PtcA and PtcB can be predicted from the phylogenetic analysis, which coincides with the results reported recently by Reitz et al. (2019). In contrast to the analysis of Reitz et al., we chose to analyze hydroxylases producing β-OH-Asn, as in the synthesis of curacomycin (Kaweewan et al., 2017) and ulleungmycin (Son et al., 2017), and β-OH-Glu, as in the synthesis of kutzneride (Strieker et al., 2009), as well as β-OH-Asp, as found in non-siderophore compounds such as malacidin A (Hover et al., 2018) and syringomycin (Gross and deVay, 1977). The phylogenetic analysis revealed that the prediction was meaningful only when the hydroxylases in the BGCs of siderophores and phytotoxins were taken into account. To better understand this phenomenon, we reconstructed the phylogenetic tree based on the 16S rRNA of the producing strains listed in Table S3 and Figure S30. It seems that the phylogenic analysis based on β-hydroxylases protein sequences (Figure 6) are likely to emphasize genus-specific mutational evolution rather than functional evolution. Although the phylogeny reported by Reitz *et al* and in this study can be used to illuminate the stereochemistry of β-hydroxylases in related genera, a more detailed phylogenetic analysis is needed to clarify the stereospecificity of β-hydroxylases derived from wider evolutionary origins.

## Data Availability Statement

The datasets generated for this study can be found in NCBI: genomic data, accession no. WJPH00000000 (https://www.ncbi.nlm.nih.gov/nuccore/WJPH00000000) and 16S rRNA sequence, accession no. MN636765 (https://www.ncbi.nlm.nih.gov/nuccore/MN636765).

## Author Contributions

YX and BH initiated and oversaw all research. GZ provided the isolated extremophiles. NH cultured all strains. LL performed the screening and potashchelin isolation. YL performed Marfey's analysis and partial hydrolysis. NH and WG extracted genomic DNA and carried out bioassays. YX performed data analysis, structure elucidation, genome assembly, and bioinformatics analysis. YX and YL co-drafted the manuscript. BH improved the manuscript.

### Conflict of Interest

The authors declare that the research was conducted in the absence of any commercial or financial relationships that could be construed as a potential conflict of interest.
