# Supplementary material for: Potashchelins, a Suite of Lipid Siderophores Bearing Both L-threo and L-erythro Beta-Hydroxyaspartic Acids, Acquired From the Potash-Salt-Ore-Derived Extremophile Halomonas sp. MG34
Source: Front Chem. 2020 Mar 20;8:197. doi: 10.3389/fchem.2020.00197 (PMC7100376; doi:10.3389/fchem.2020.00197)
Supplement: Supplementary file 1 [file Data_Sheet_1.PDF]

Supplementary Material

# Potashchelins, a suite of lipid siderophores bearing both L-*threo* and L-*erythro* beta-hydroxyaspartic acids, acquired from the potash-salt-ore-derived extremophile *Halomonas* sp. MG34

**Yihong Li[1†], Li Liu[1†‡], Gengxin Zhang[3], Ning He[1], Wenqiang Guo[1§], Bin Hong[1, 2*], Yunying Xie[1*]**

[1]CAMS Key Laboratory of Synthetic Biology for Drug Innovation, [2]NHC Key Laboratory of Biotechnology of Antibiotics, Institute of Medicinal Biotechnology, Chinese Academy of Medical Sciences & Peking Union Medical College, Tiantan xili No.1, Beijing 100050, China.
[3]Key Laboratory of Alpine Ecology, Institute of Tibetan Plateau Research, China Academy of Sciences, Beijing 100101, China

*** Correspondence:**
Yunying Xie
xieyy@imb.pumc.edu.cn (Yunying Xie)

Bin Hong
hongbin@imb.pumc.edu.cn (Bin Hong)

[†] These authors contributed equally to this work.
[‡] Present address: Institute of Chinese Materia Medica, China Academy of Chinese Medical Sciences, Beijing 100700, China
[§] Department of Medicine, Jinggangshan University, Ji'an, Jiangxi 343009, China

## 1. Supplementary Figures

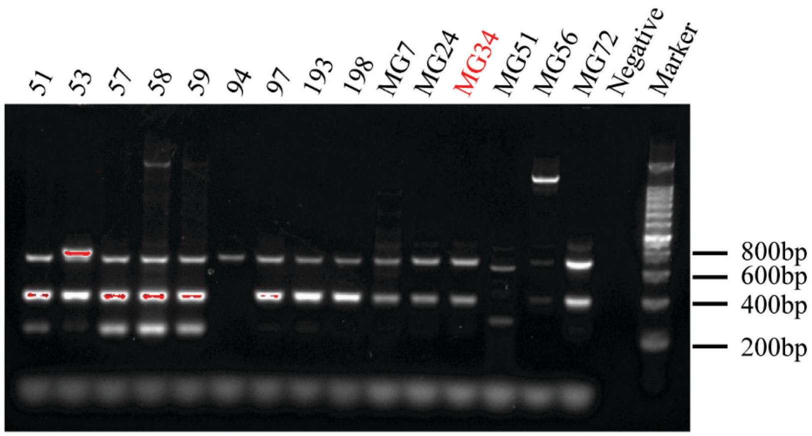


**Figure S1.** The Agarose gel electrophoresis analysis of PCR products for screening NRPs producers. The potential NRPS adenylation sequences were amplified using genomic DNAs of 15 halophile strains as templates and A3F/A7R as primers. The size of expected PCR products is 700-800 bp.

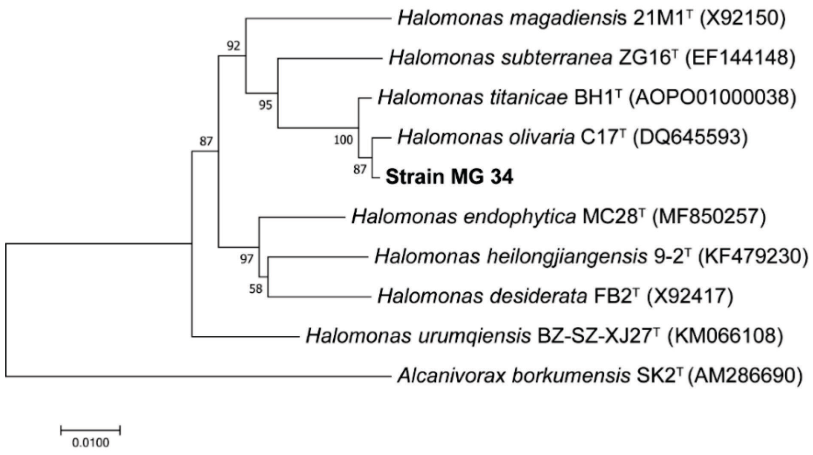


**Figure S2.** The phylogenetic NJ tree based on 16S rRNA gene sequences from strain MG34 and its homologs. The sequence of 16S rRNA of *Alcanivorax borkumensis* SK2 was used as an outgroup. The GenBank accessions are shown in parentheses.

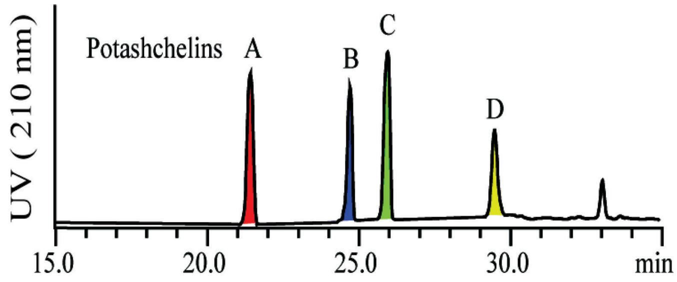


**Figure S3.** HPLC spectrum of potashchelins A-D. The fifth peak with a quasi-molecular ion $[M+H]^+$ at *m/z* 1125 is an artificial product of potashchelins D, which was produced during the isolation progress by opening of the ring of cyclic N($\delta$)-hydroxyornithine (data not shown).

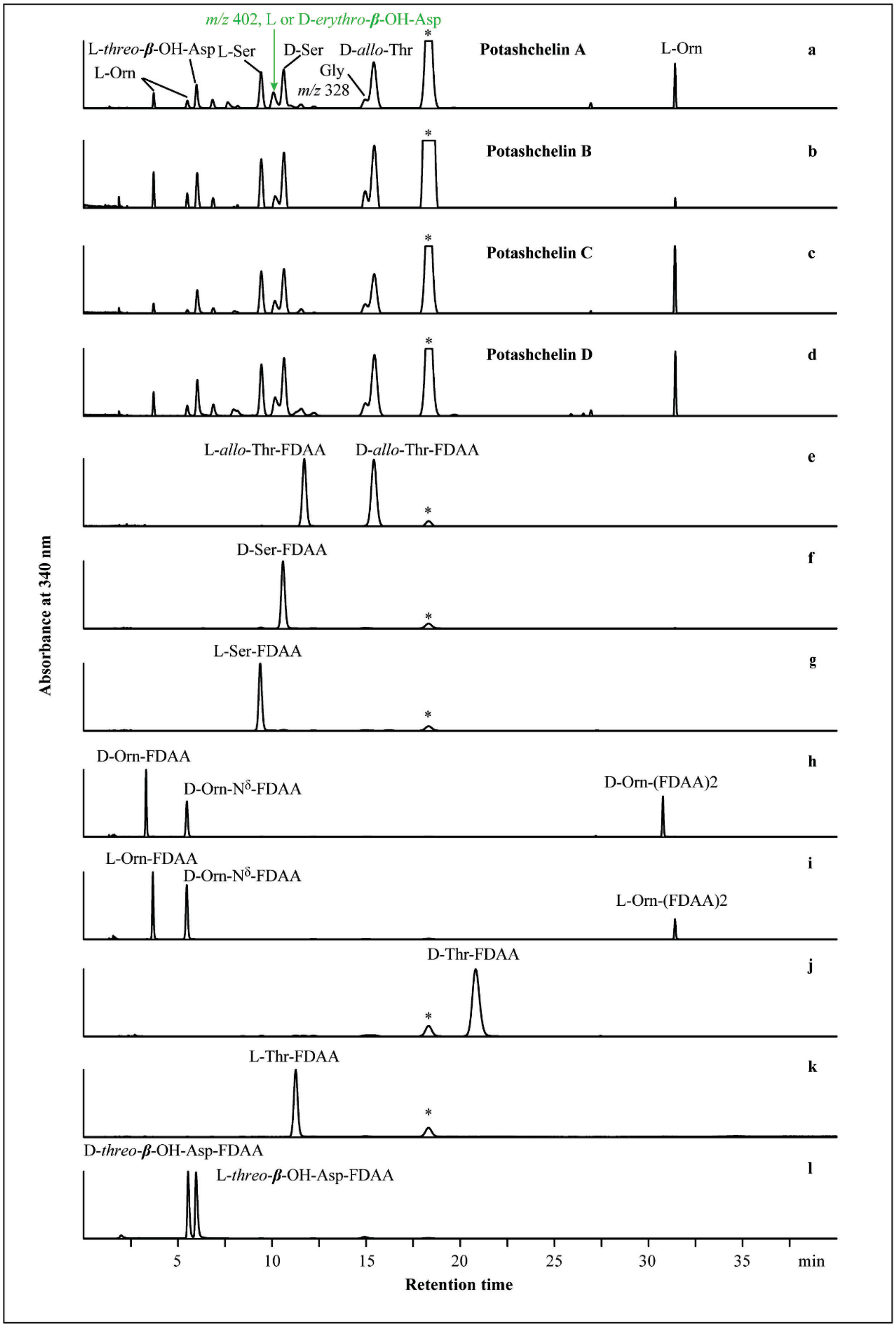


**Figure S4.** $C_3$ Marfey's analysis for potashchelins A-D (**1**-**4**). **a**-**d**: HPLC-DAD (340 nm) chromatograms of hydrolysate of **1**-**4** derivatized with L-FDAA, respectively. **f**-**l**: HPLC-DAD (340 nm) chromatograms of L-FDAA derivatized standards DL-*allo*-Thr, D-Ser, L-Ser, D-Orn, L-Orn, D-Thr, L-Thr, DL-*threo*-*β*-OH-Asp, separately. * Residual L-FDAA

A

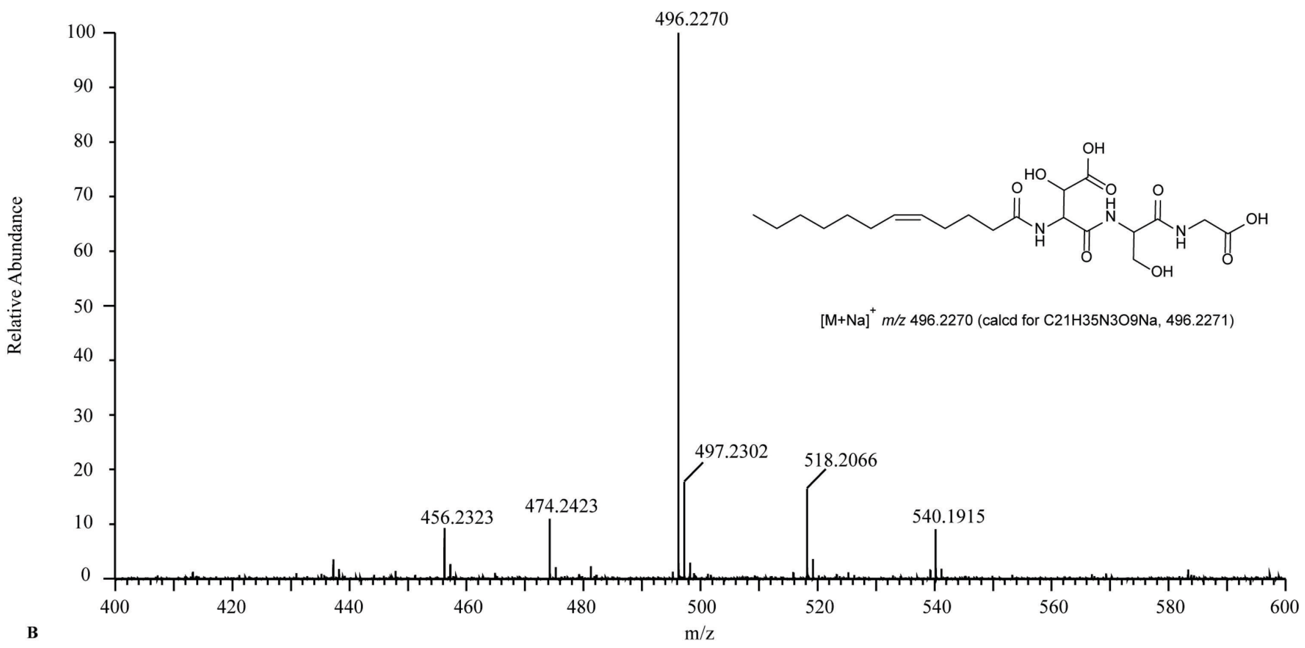


B

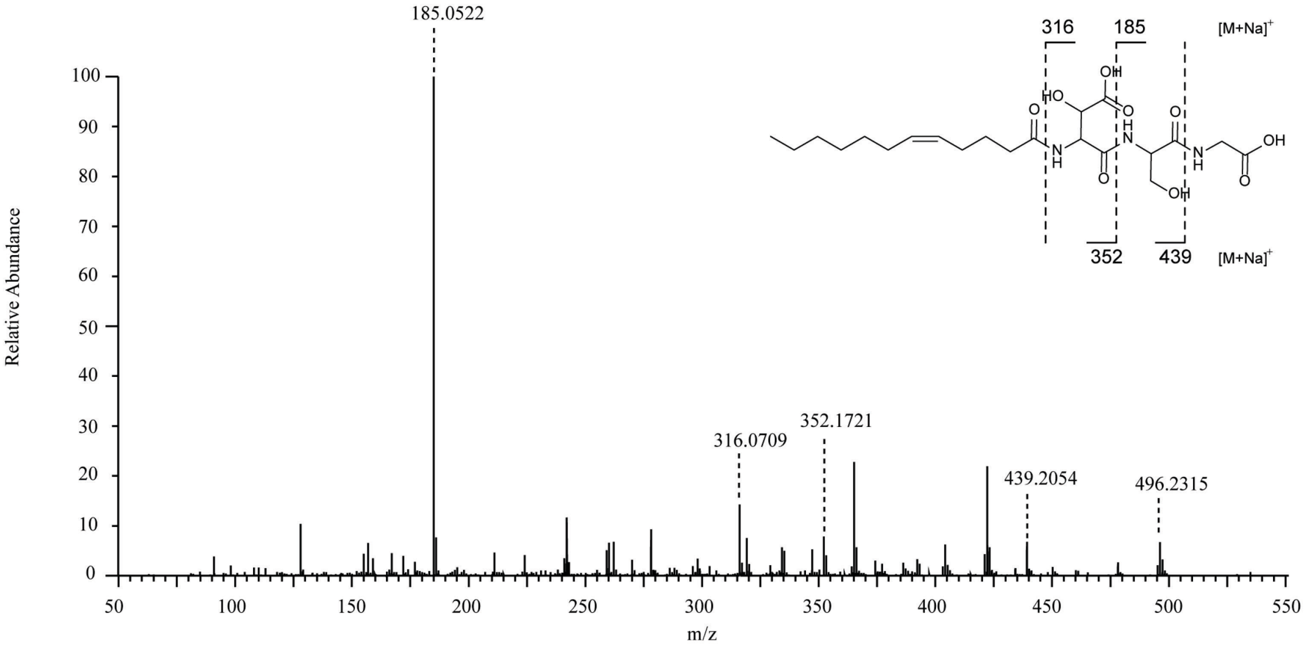


**Figure S5.** ESI-MS and MS/MS spectra for partial hydrolysate of potashchelin B

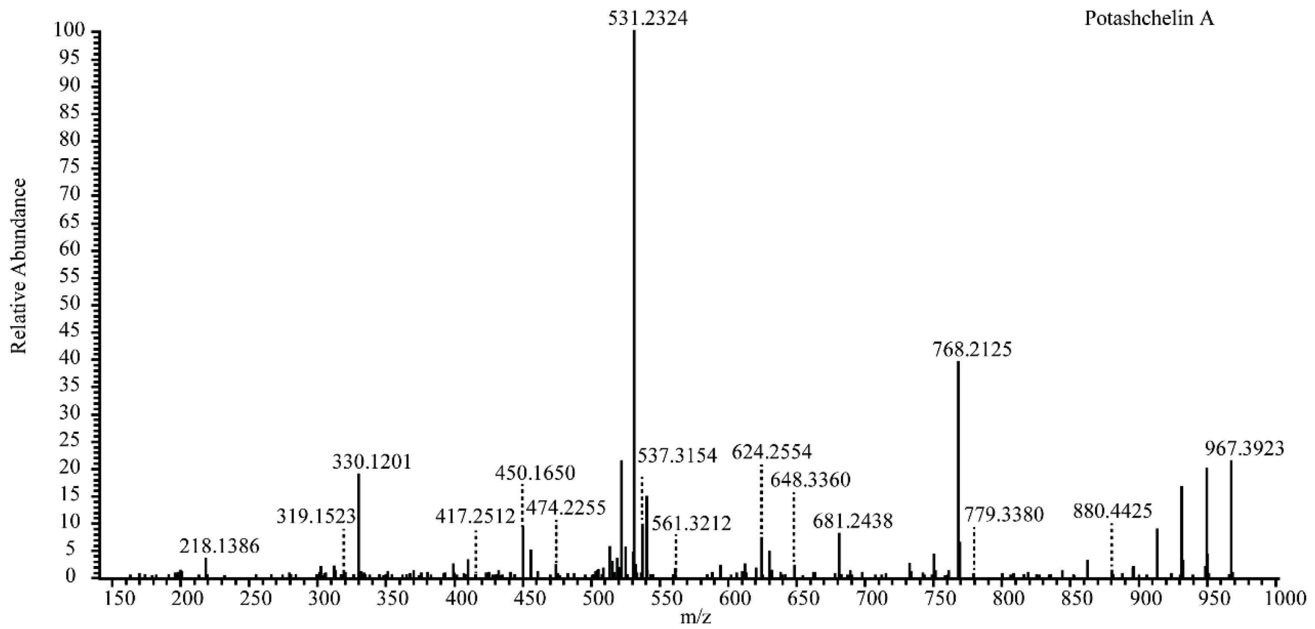


**Figure S6.** ESI-MS/MS fragmentation of potashchelin A

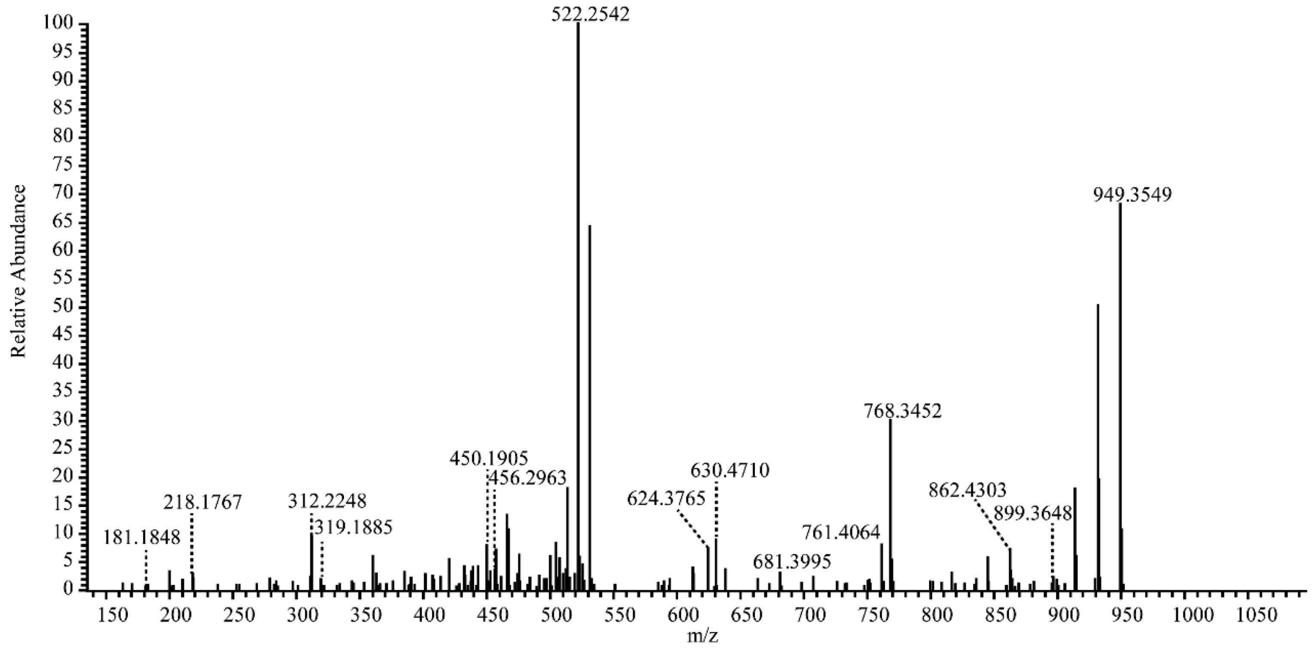


**Figure S7.** ESI-MS/MS fragmentation of potashchelin B

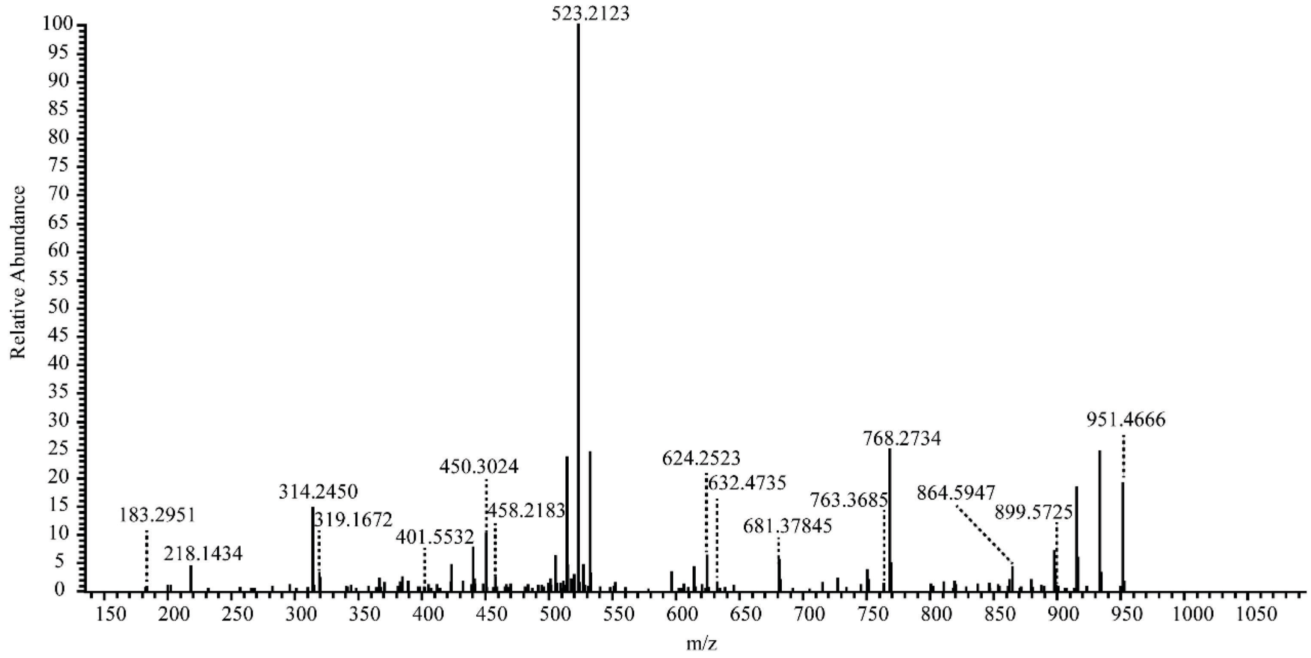


**Figure S8.** ESI-MS/MS fragmentation of potashchelin C

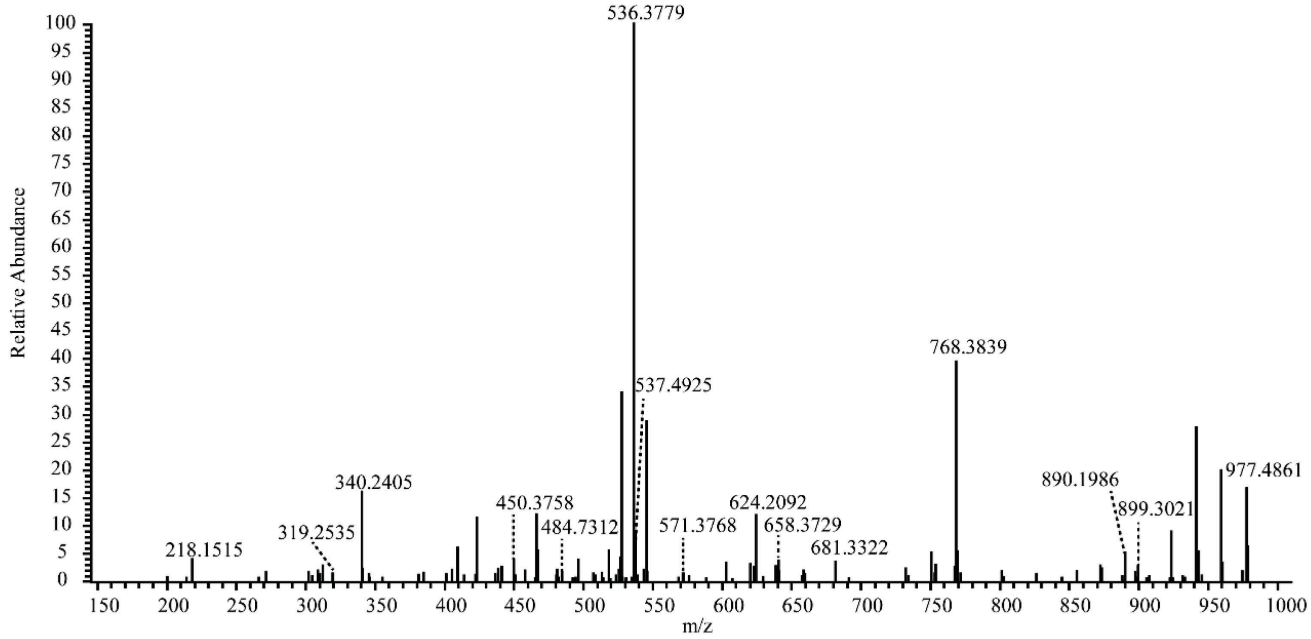


**Figure S9.** ESI-MS/MS fragmentation of potashchelin D

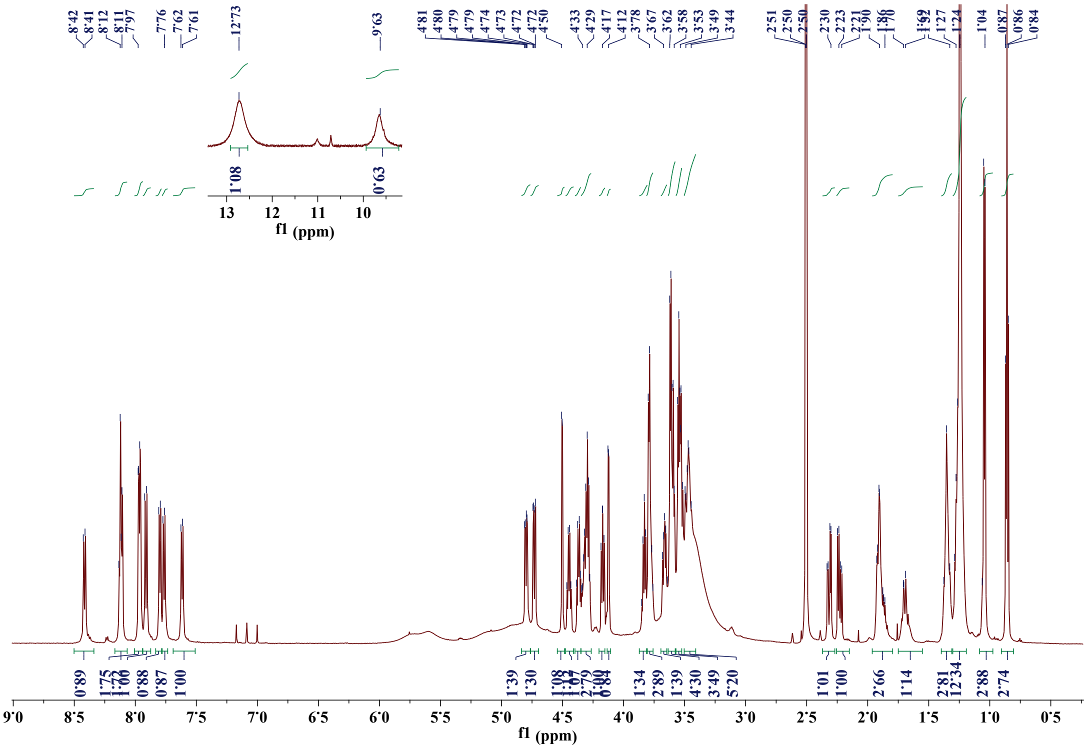


**Figure S10.** $^{1}$H NMR for **1** (potashchelin A) in DMSO-$d_6$ (600 MHz)

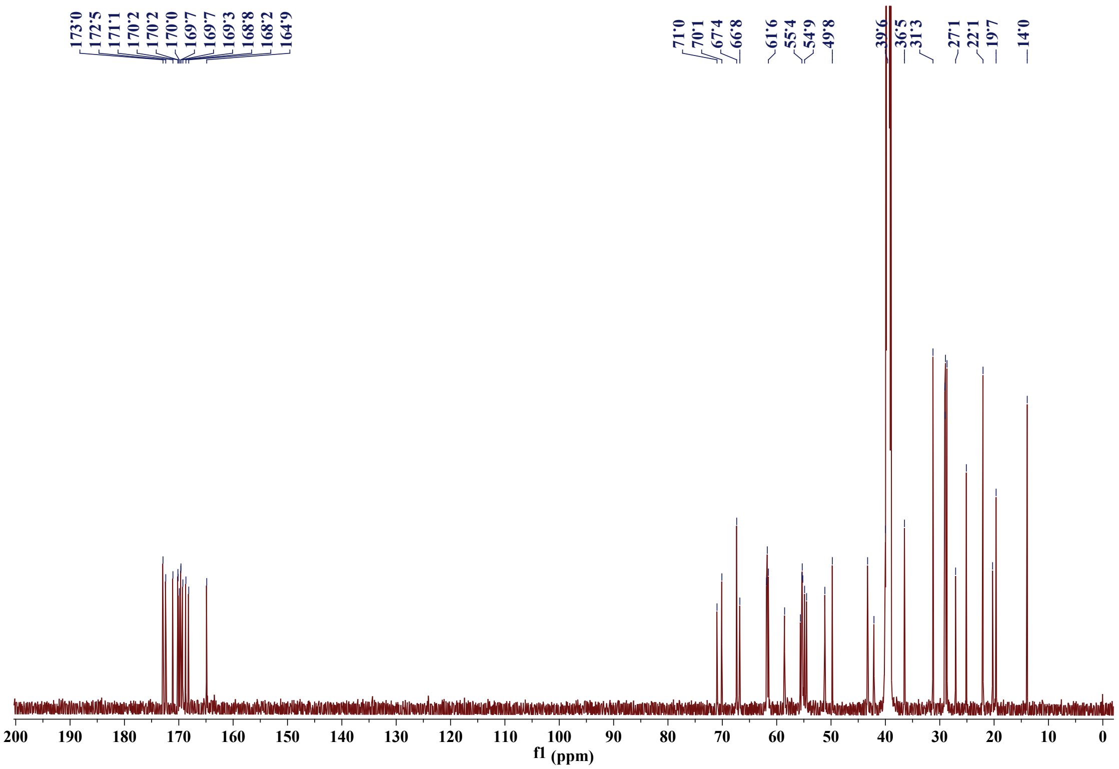


**Figure S11.** $^{13}$C NMR for **1** (potashchelin A) in DMSO-$d_6$ (150 MHz)

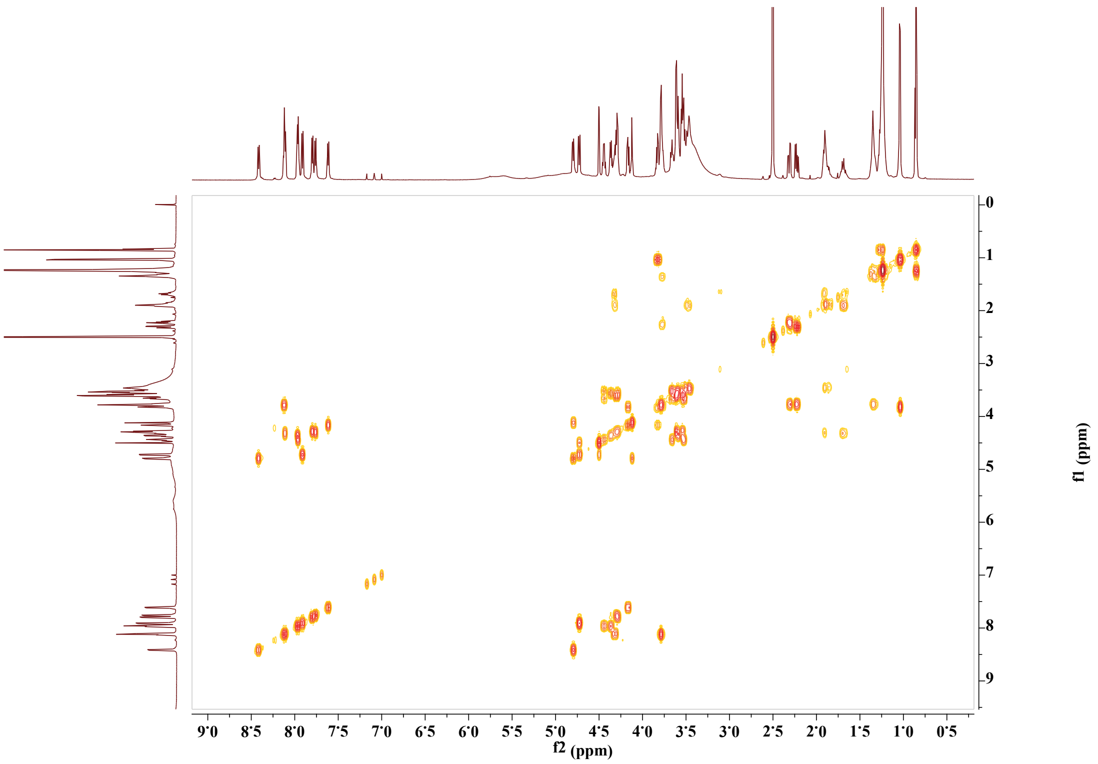


**Figure S12.** gCOSY for **1** (potashchelin A) in DMSO-$d_6$

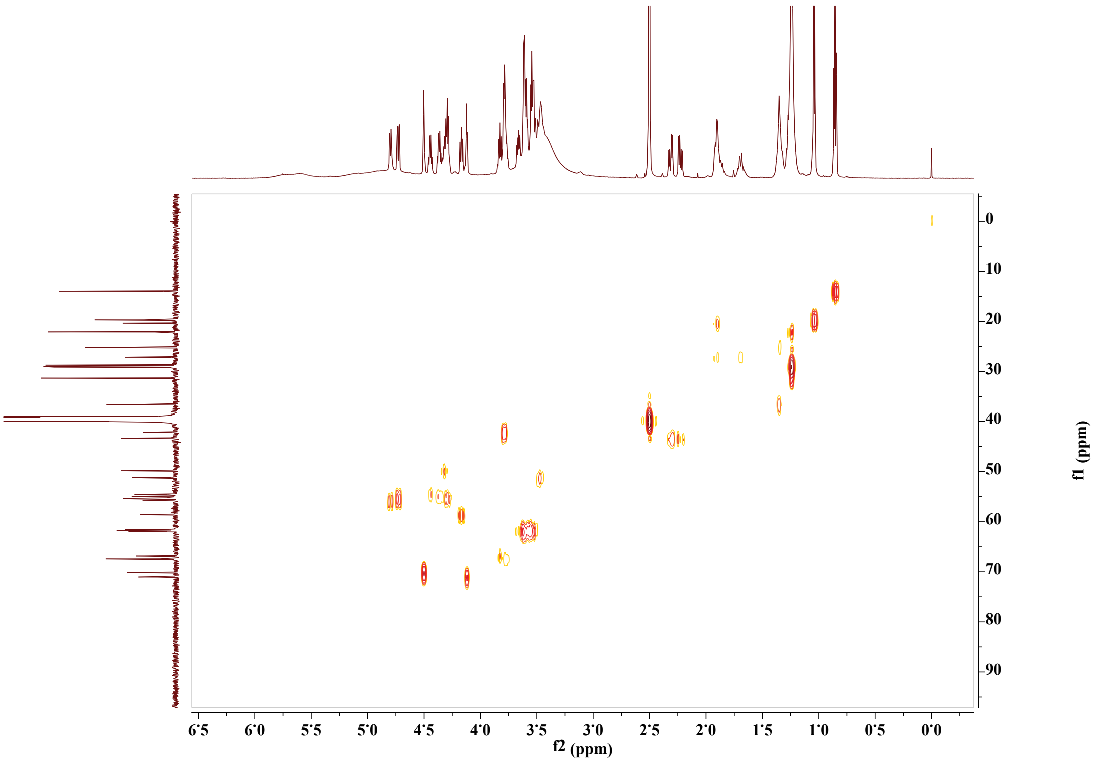


**Figure S13.** HSQC for **1** (potashchelin A) in DMSO-$d_6$

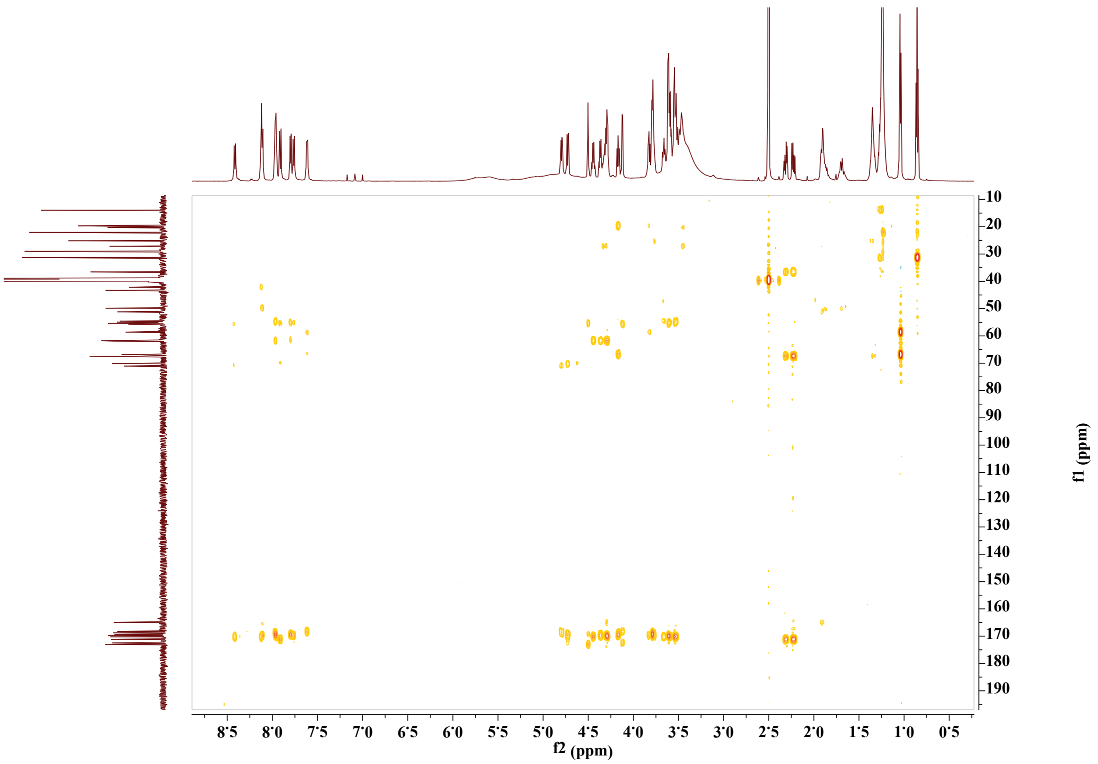


**Figure S14.** HMBC for **1** (potashchelin A) in DMSO-$d_6$

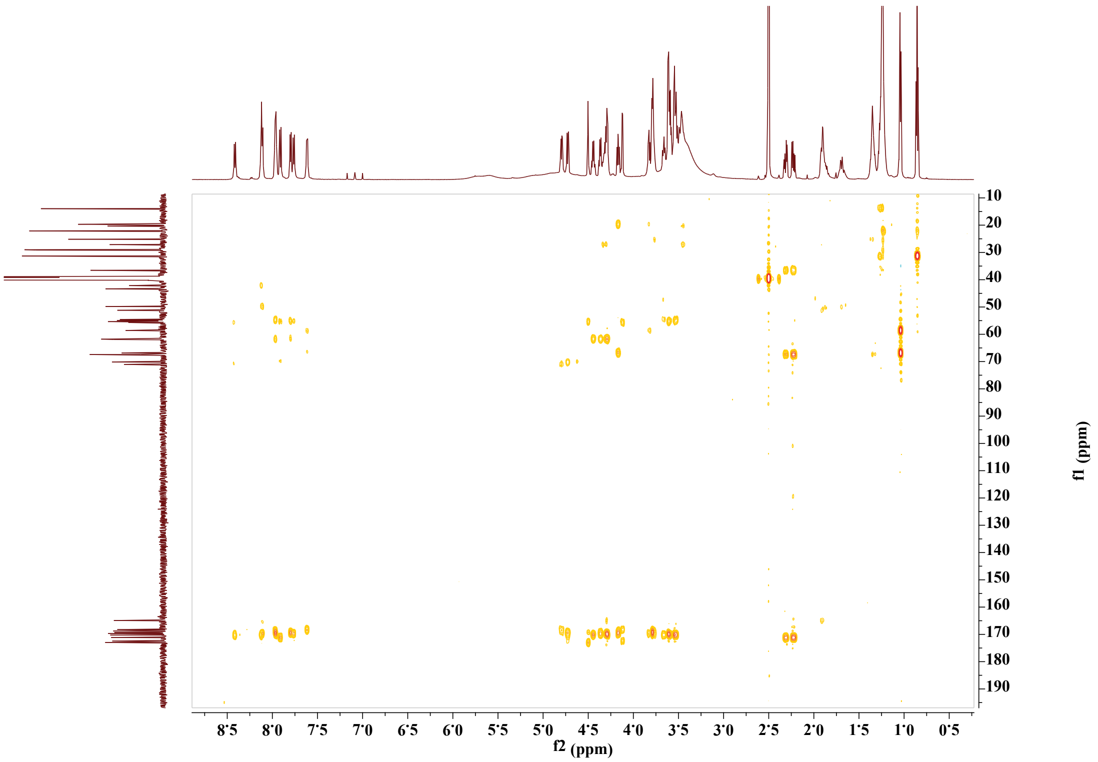


**Figure S15.** HMBC for **1** (potashchelin A) in DMSO-$d_6$

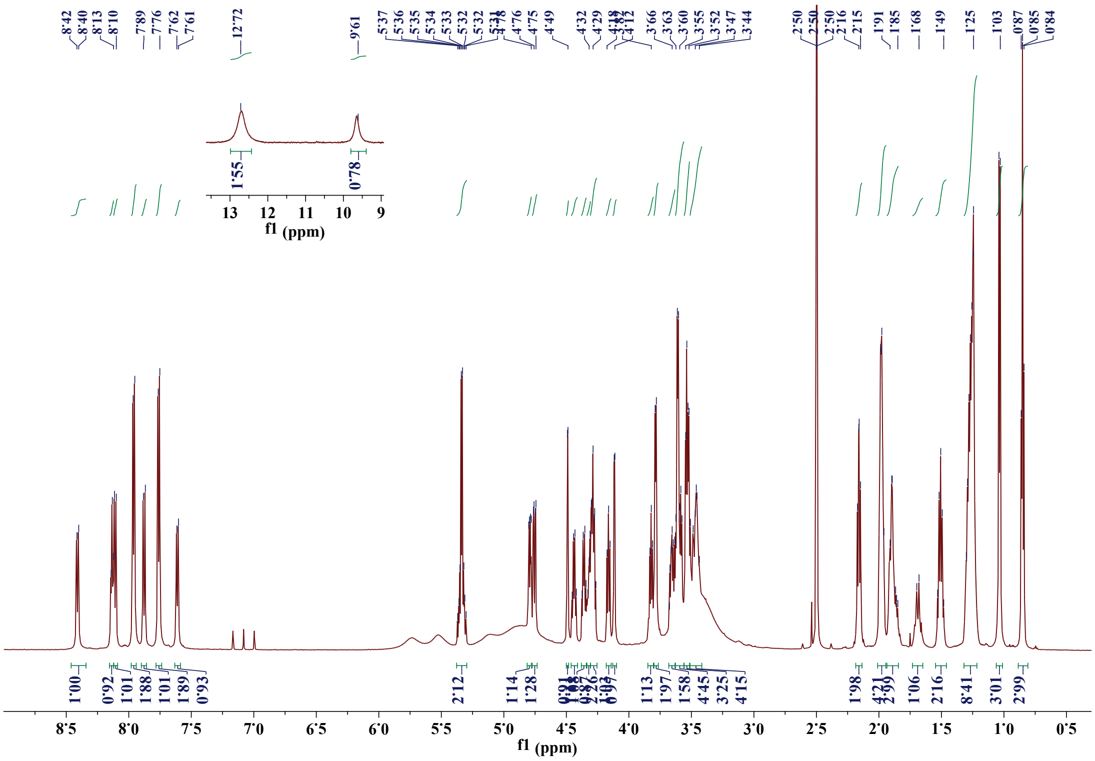

**Figure S16.** $^{1}H$ NMR for **2** (potashchelin B) in DMSO-$d_6$ (600 MHz)

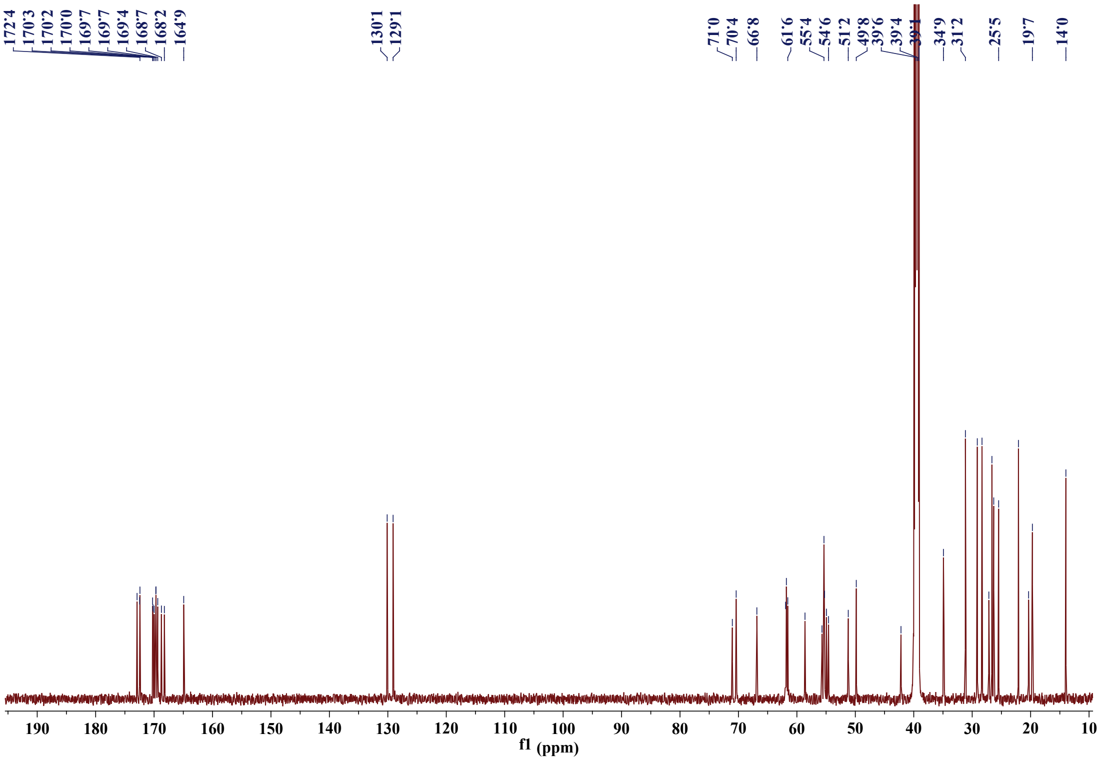

**Figure S17.** $^{13}C$ NMR for **2** (potashchelin B) in DMSO-$d_6$ (150 MHz)

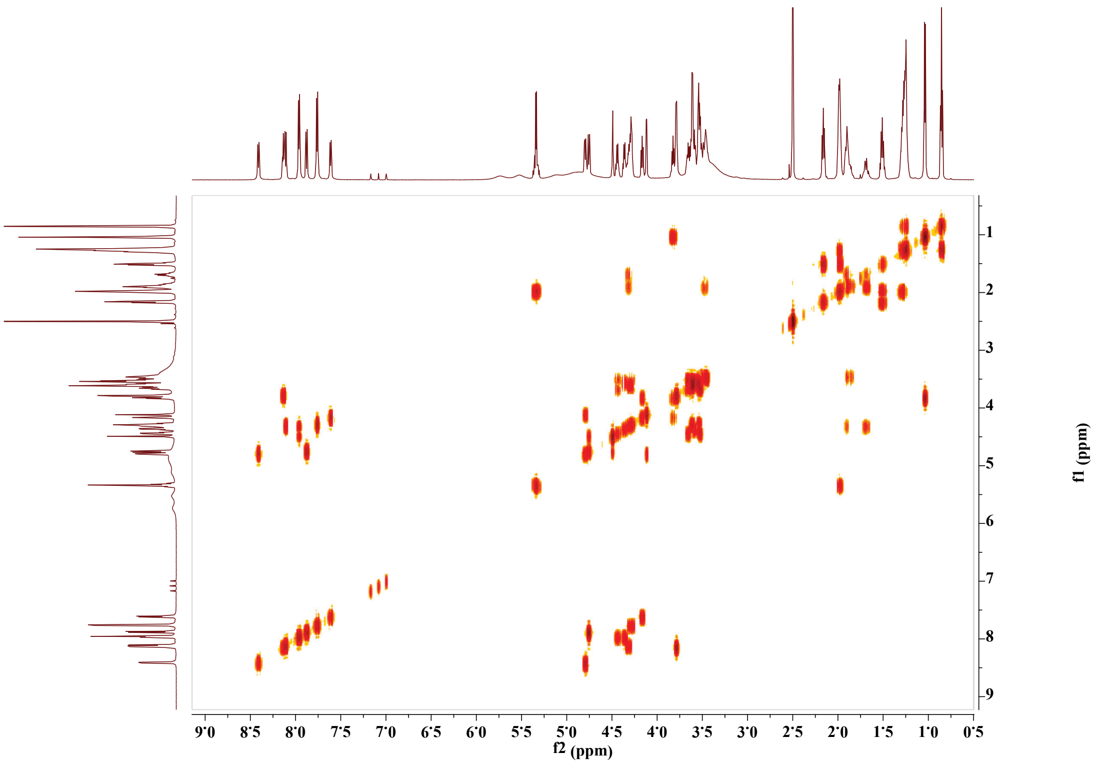


**Figure S18.** gCOSY for **2** (potashchelin B) in DMSO-$d_6$

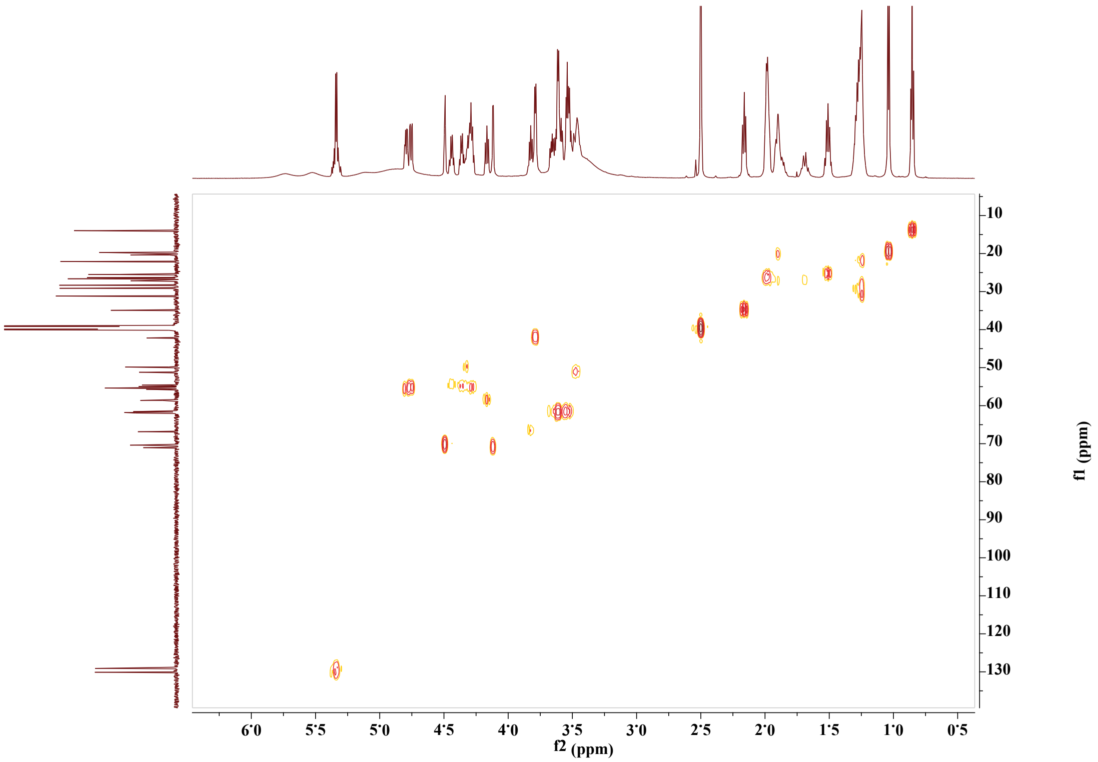


**Figure S19.** HSQC for **2** (potashchelin B) in DMSO-$d_6$

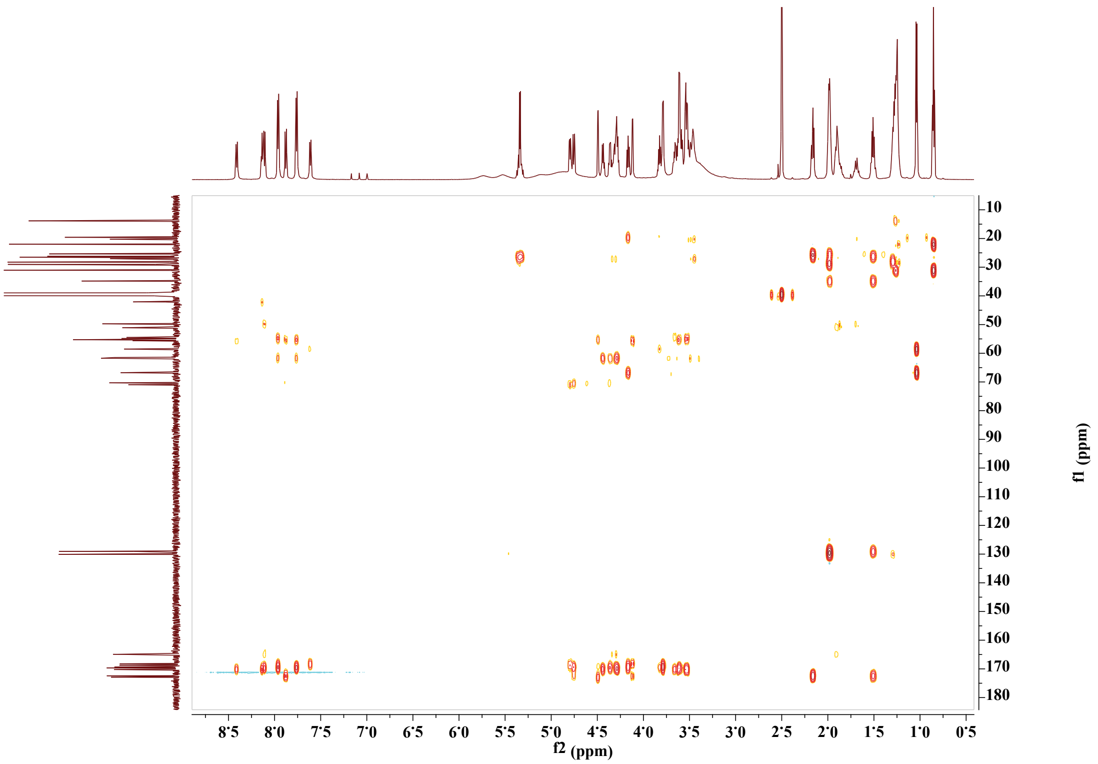


**Figure S20.** HMBC for **2** (potashchelin B) in DMSO-$d_6$

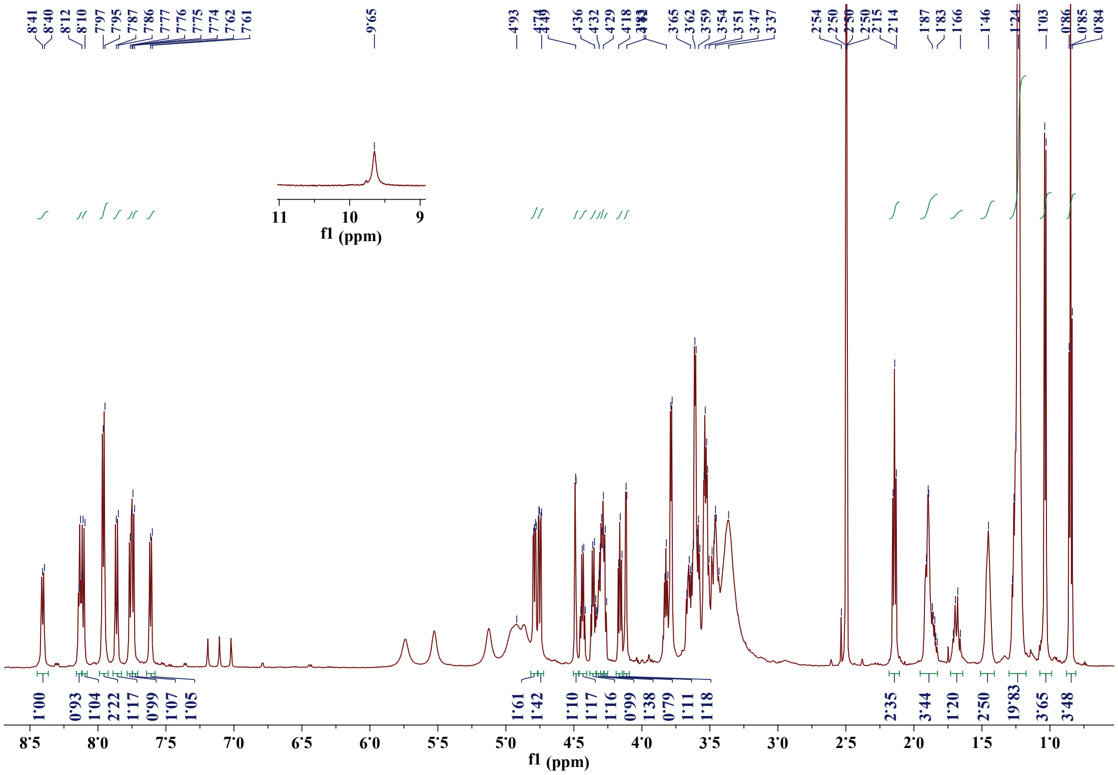


**Figure S21.** $^1$H NMR for **3** (potashchelin C) in DMSO-$d_6$ (600 MHz)

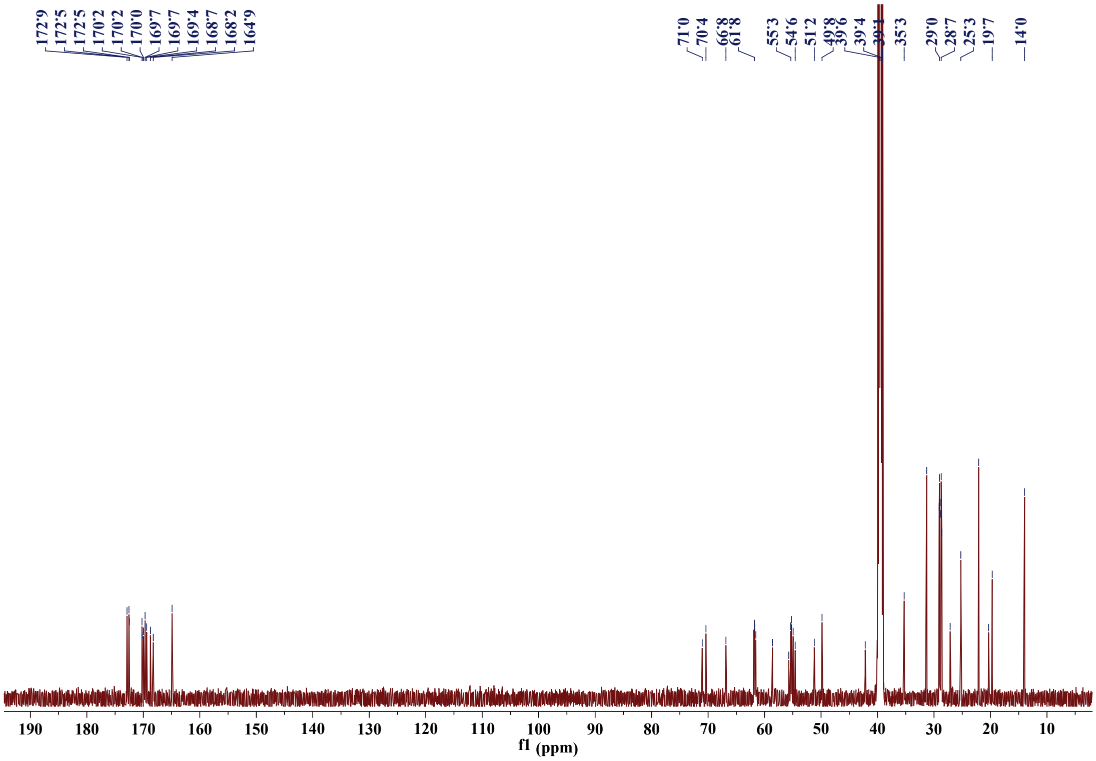


**Figure S22.** $^{13}C$ NMR for **3** (potashchelin C) in DMSO-$d_6$ (150 MHz)

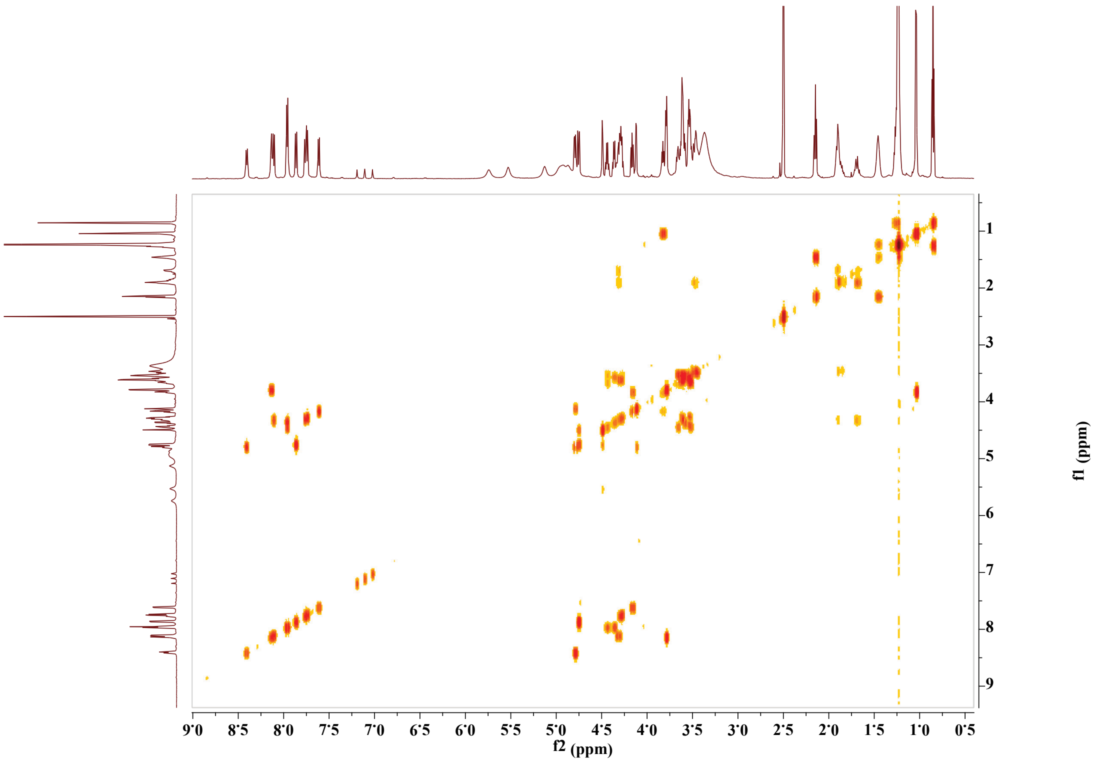


**Figure S23.** gCOSY for **3** (potashchelin C) in DMSO-$d_6$

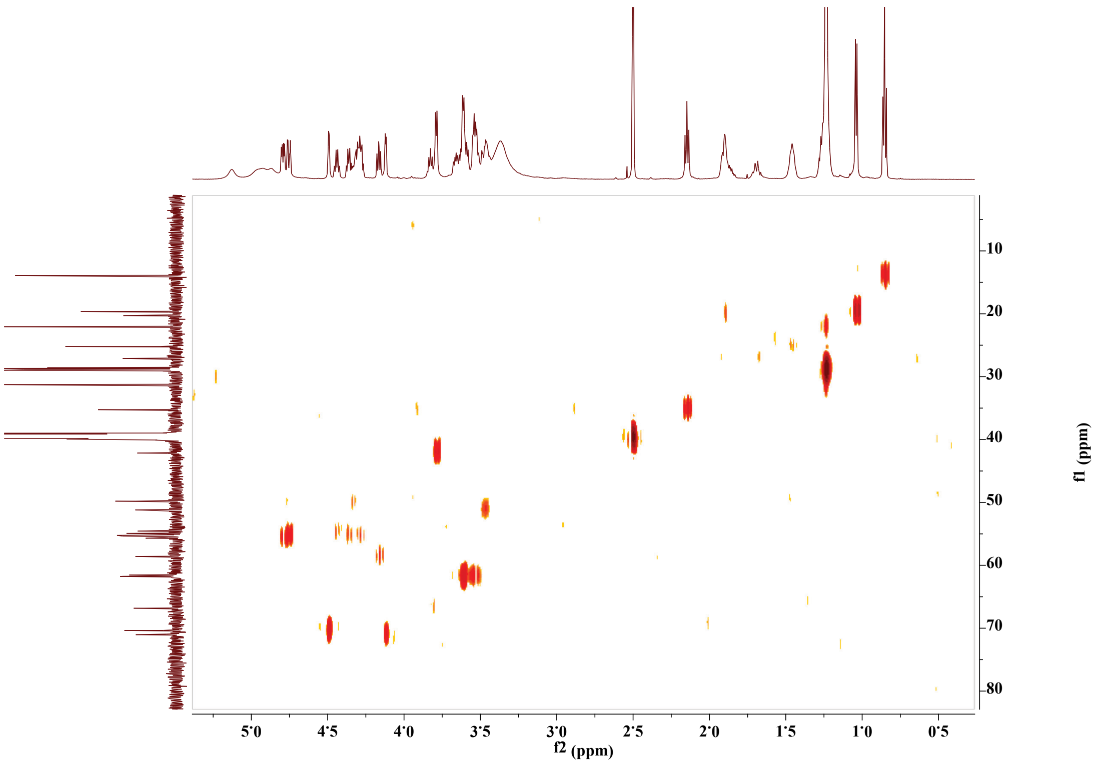


**Figure S24.** HSQC for **3** (potashchelin C) in DMSO-$d_6$

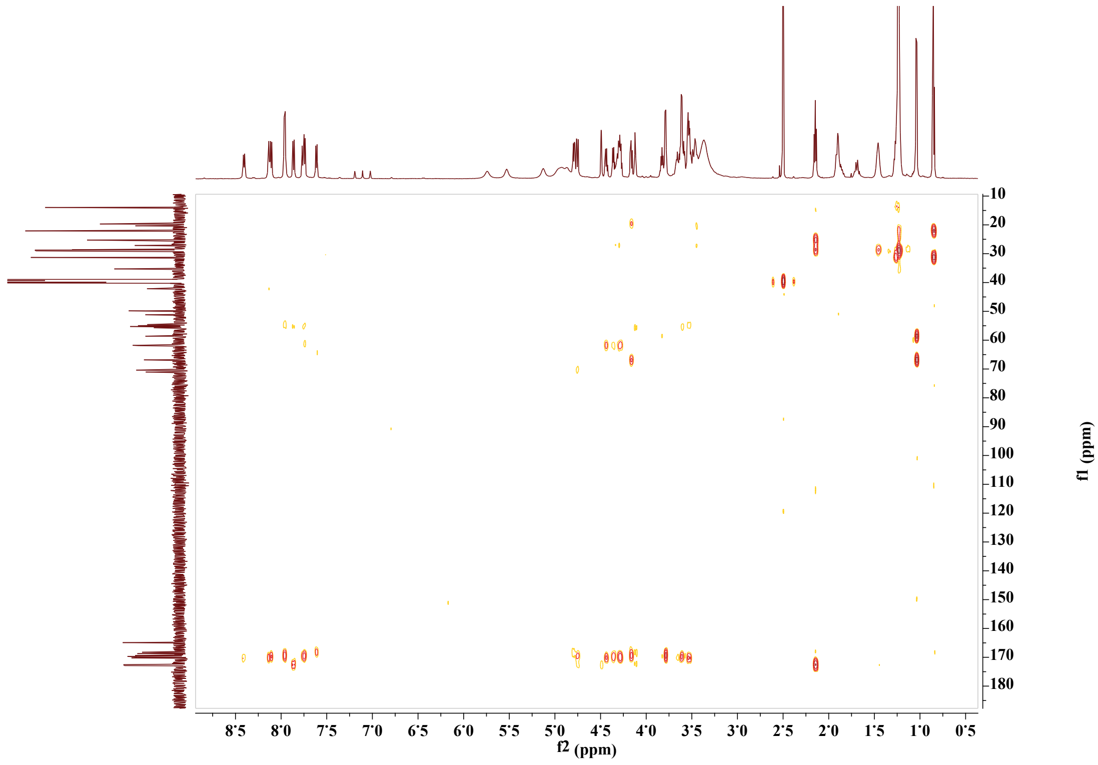


**Figure S25.** HMBC for **3** (potashchelin C) in DMSO-$d_6$

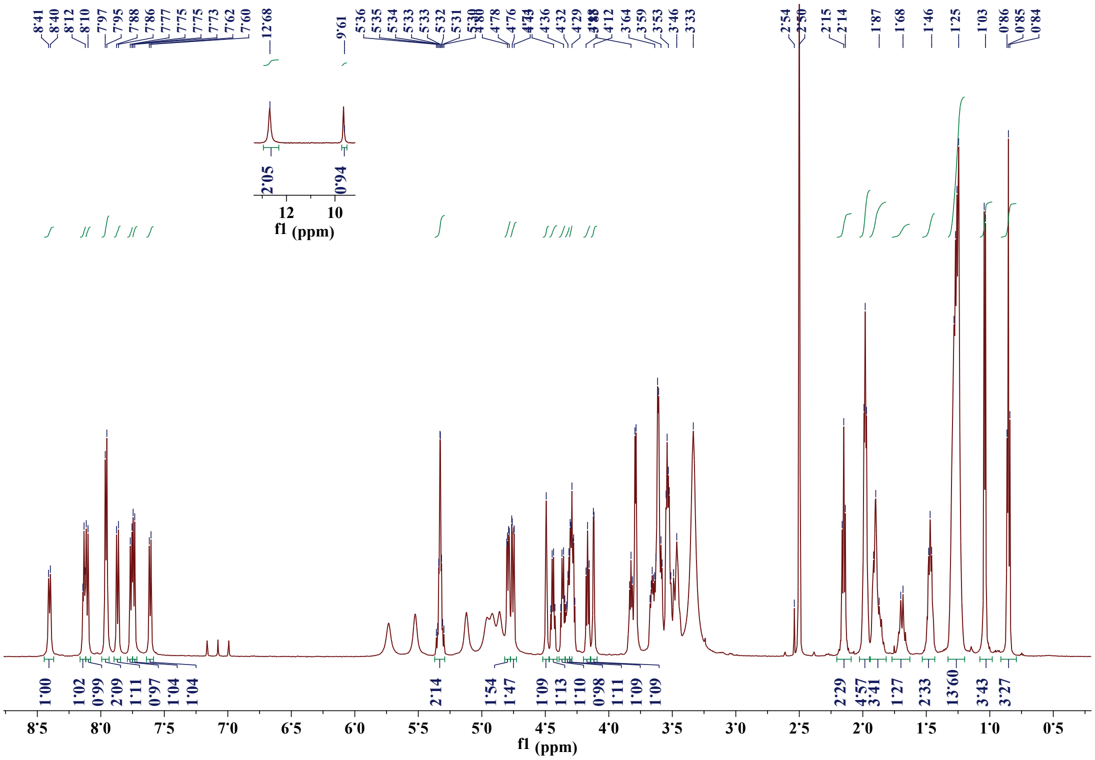


**Figure S25.** $^{1}H$ NMR for **4** (potashchelin D) in DMSO-$d_6$ (600 MHz)

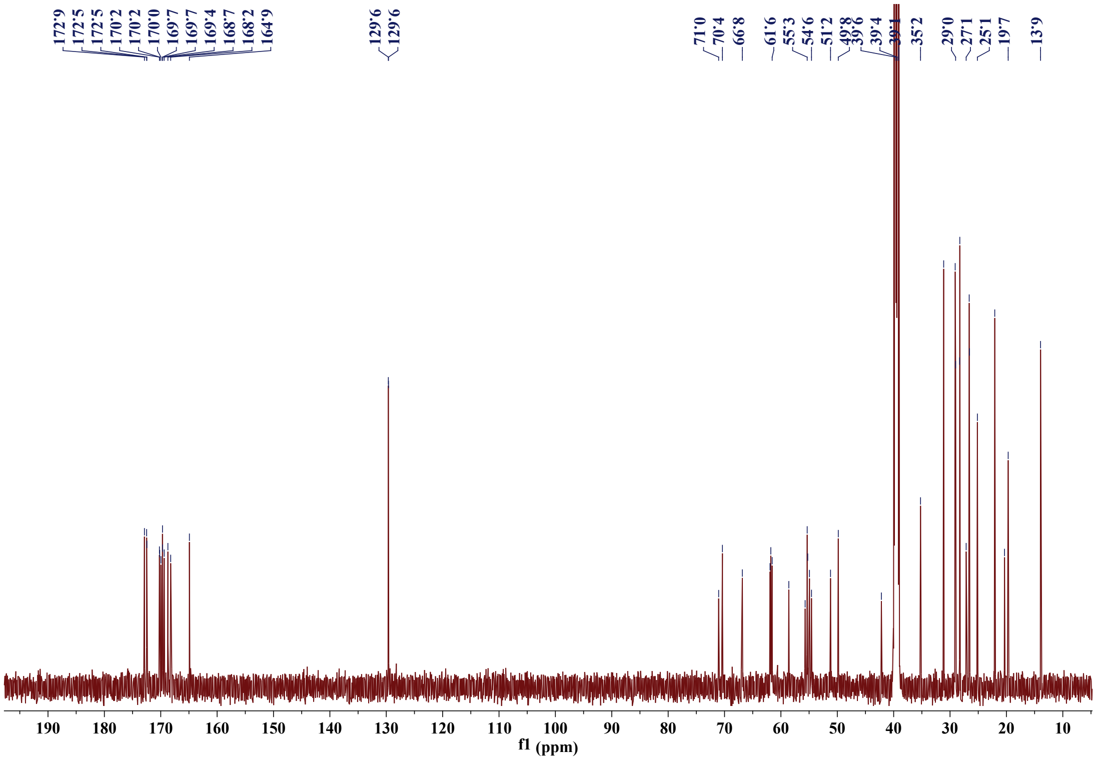


**Figure S26.** $^{13}C$ NMR for **4** (potashchelin D) in DMSO-$d_6$ (150 MHz)

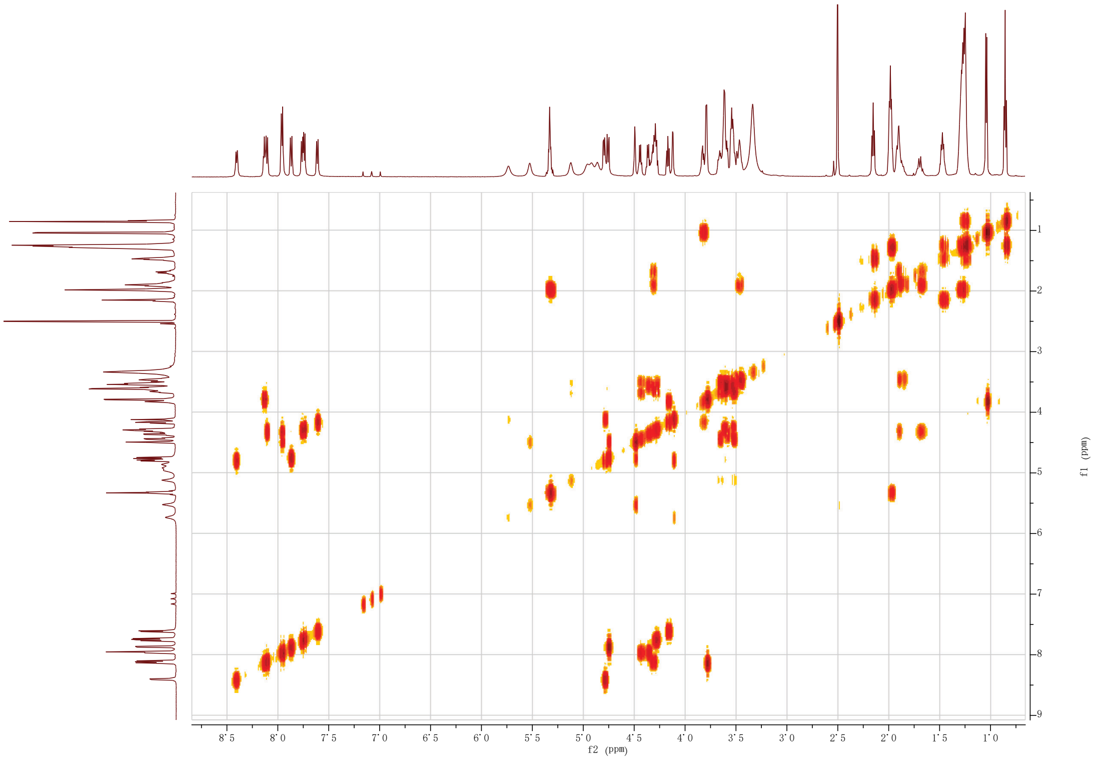


**Figure S27.** gCOSY for **4** (potashchelin D) in DMSO-$d_6$

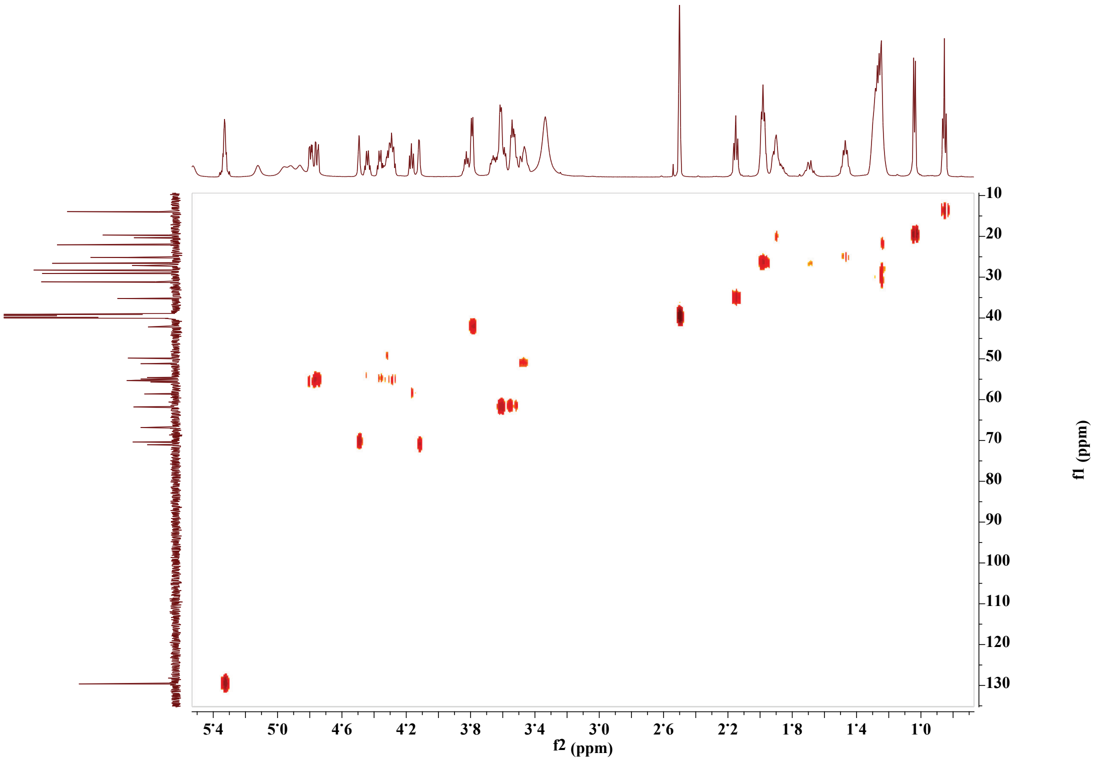


**Figure S28.** HSQC for **4** (potashchelin D) in DMSO-$d_6$

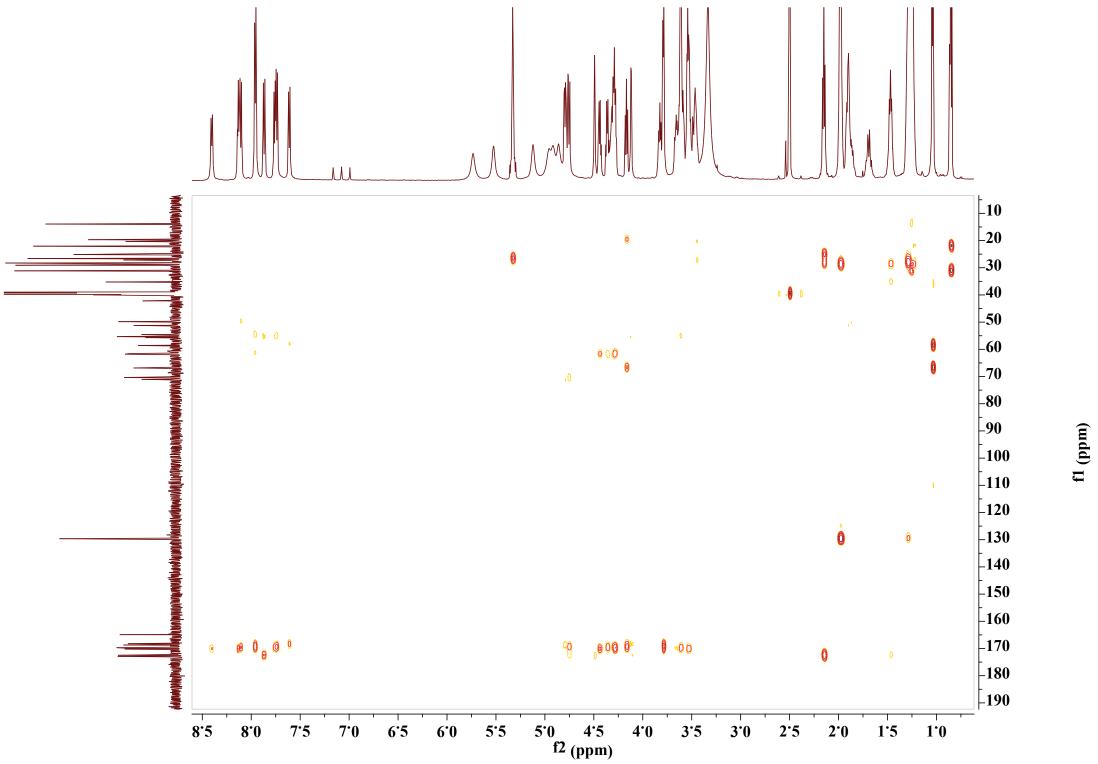


**Figure S29.** HMBC for **4** (potashchelin D) in DMSO-$d_6$

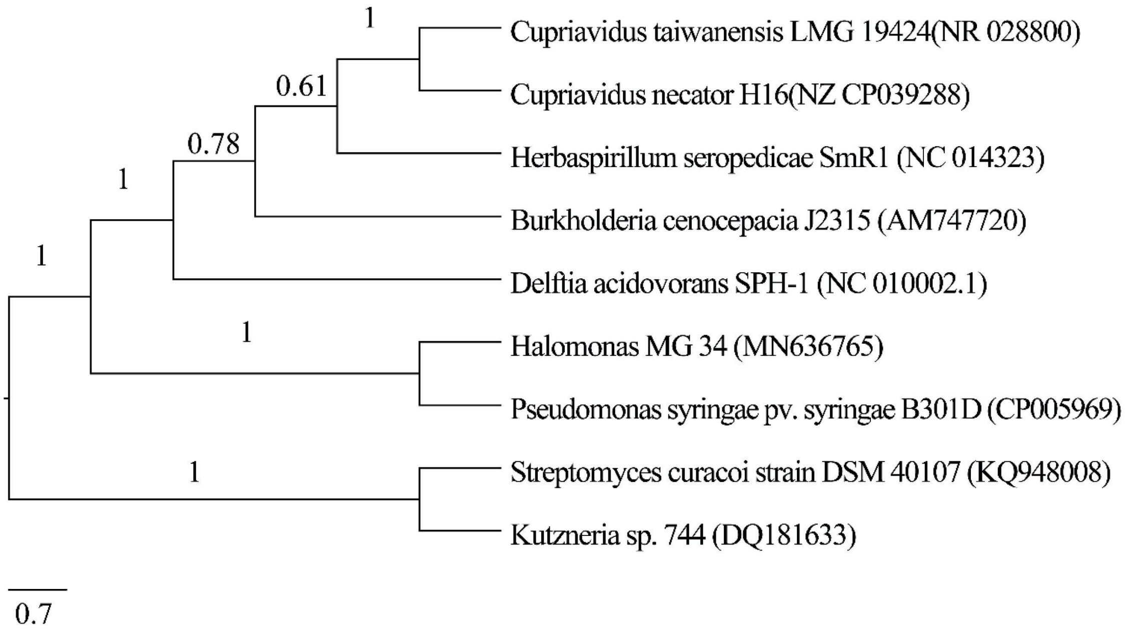


**Figure S30.** The phylogenetic NJ tree based on 16S rRNA gene sequences from the producing strains listed in Table S3 (16S rRNA gene sequences *of Streptomyces* sp. KCB13F003, *Streptomyces viridochromogenes* ATCC 29814 and *Actinoplanes firuliensis* were not obtained from NCBI). The GenBank accessions are shown in parentheses.

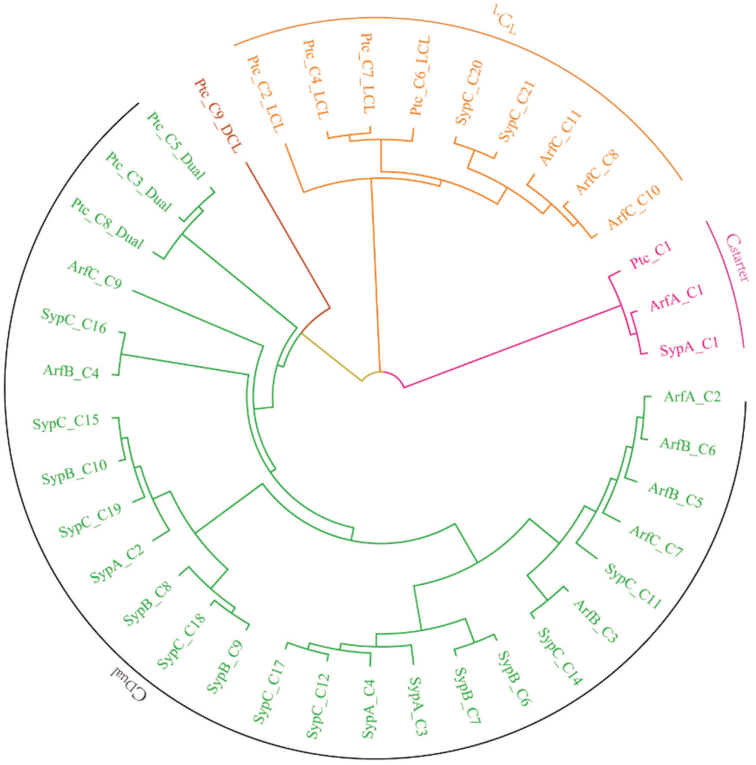


**Figure S31.** Phylogenic tree of C domains from Potashchlins (*Ptc*), Arthrofactin (*Arf*) (Balibar et al., 2005), and Syringopeptin (*Syp*) (Scholz-Schroeder et al., 2003) biosynthesis gene clusters. Evolutionary analyses were conducted in MEGA7 (Kumar et al., 2016) using the Neighbor-Joining method (Saitou and Nei, 1987).

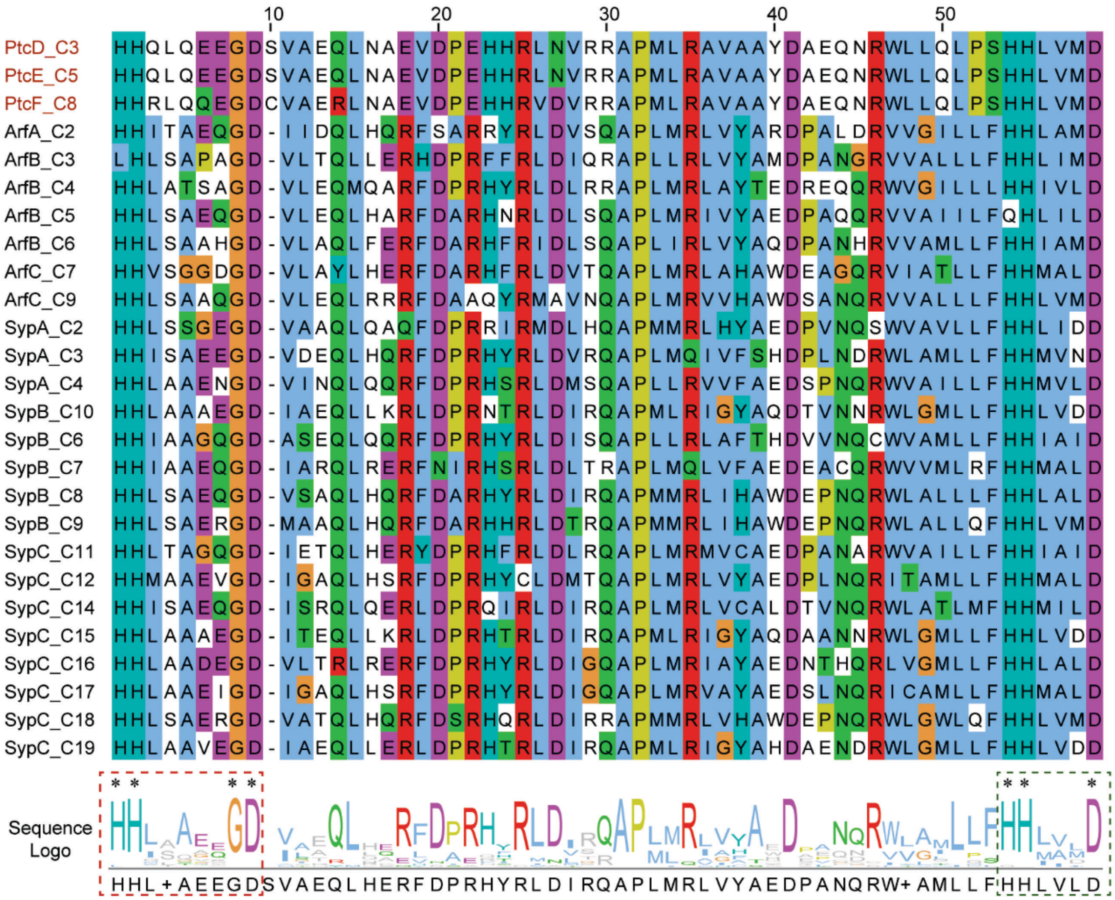

**Figure S32.** Amino acid sequence alignment of the two His-motifs of the $C_{Dual}$ domains (Balibar et al., 2005) from Potashchlins (*Ptc*), Arthrofactin (*Arf*) (Balibar et al., 2005), and Syringopeptin (*Syp*) (Scholz-Schroeder et al., 2003). The blue square indicates the His-motif (HHxxxD) conserved in all C domains, while the red square highlighted the second His-motif (HHxxxxxGD) conserved in $C_{Dual}$ domains

*

## 2. Supplementary Tables

**Table S1.** Fermentation media used in this study

| Medium | Ingredient | Reference |
|---|---|---|
| CGY | 5 g/L bacto casitone, 5 g/L glycerol, 1 g/L yeast extract, adjusted to pH 7 | (Palomo et al., 2013) |
| CGY (+) | CGY5 medium supplemented with 30 g/L NaCl | This study |
| DEF-15 | 40 g/L sucrose, 2 g/L $NH_4Cl$, 2 g/L $Na_2SO_4$, 1 g/L $K_2HPO_4$, 1 g/L $MgCl_2 \cdot 6H_2O$, 1 g/L NaCl, 2% g/L $CaCO_3$, 1mL trace elements solution (100 mg/L $MnCl_2 \cdot 4H_2O$, 100 mg/L $ZnCl_2$, 100 mg/L $FeCl_2 \cdot 4H_2O$, 50 mg/L NaI), adjusted to pH 7) | (Palomo et al., 2013) |
| DEF-15 (+) | DEF-15 medium supplemented with 30 g/L NaCl | This study |
| IN | 2 g/L DL-serine, 2 g/L DL-alanine, 8.6 g/L $K_2SO_4$, 1.4 g/L KCl, 1.4 g/L $MgSO_4 \cdot 7H_2O$, 10 g/L sucrose, 30 g/L yeast extract, adjusted to pH 7 | (Palomo et al., 2013) |
| IN(+) | IN medium supplemented with 30 g/L NaCl | This study |
| R358 | 10 g/L starch from potato, 4 g/L yeast extract, 2 g/L peptone, 5ml KBr (20g/L), 0.5ml $FeSO_4 \cdot 7H_2O$ (8 g/L), adjusted to pH 7 | (Palomo et al., 2013) |
| R358 (+) | R358 medium supplemented with 30 g/L NaCl | This study |
| ISP2 | 20 g/L yeast extract, 10 g/L maltose, 4 g/L glucose | (Shirling and Gottlieb, 1966) |
| ISP2 (+) | ISP2 medium supplemented with 30 g/L NaCl | This study |
| AM | 10 g/L tryptone, 5 g/L yeast extract, 0.5 g/L glycine | (Bitzer et al., 2006) |
| AM (+) | AM medium supplemented with 30 g/L NaCl | This study |
| S5 (-) | Culture medium S5 deprived of agar | This study |

**Table S2.** The antibacterial activities of the strain MG 34

| Fermentation Media | *B. subtilis* CMCC 100027 | *M. phlei* CMCC 160023 | *S. aureus* ATCC 29213 | *E. coli* ATCC 25922 | *P. aeruginosa* ATCC 27853 | *C. albicans* ATCC 10231 |
|---|---|---|---|---|---|---|
| CGY (+) | - | - | - | - | - | - |
| CGY | - | - | - | - | - | - |
| DEF-15 (+) | + | - | + | - | - | - |
| DEF-15 | - | - | - | - | - | - |
| IN (+) | - | - | - | - | - | - |
| IN | - | - | - | - | - | - |
| R358 (+) | - | - | - | - | - | - |
| R358 | - | - | - | - | - | - |
| ISP2 (+) | - | - | - | - | - | - |
| ISP2 | - | - | - | - | - | - |
| AM (+) | - | - | - | - | - | - |
| AM | - | - | - | - | - | - |
| S5(-) | - | - | - | - | - | - |

+: exhibiting activity; -: No detectable activity

**Table S3.** MiBIG-provided homologs of PtcA involved in the beta-hydroxylation of Asp, Glu, and Asn

| Compounds | Producing strain | Hydroxylase | Protein Accessions | Products of Hydroxylase | Absolute stereo of C3 | References |
|---|---|---|---|---|---|---|
| Potashchelins | *Halomonas* sp. MG 34 | PtcA | NAO96315 | L-*erythro*-*β*-OH-Asp | R | This study |
| | | PtcB | NAO96316 | L-*threo*-*β*-OH-Asp | S | This study |
| Taiwachelin | *Cupriavidus taiwanensis* LMG 19424 | TaiD | YP_002007883 | L-*threo*-*β*-OH-Asp | S | (Kreutzer and Nett, 2012) |
| Cupriachelin | *Cupriavidus necator* H16 | CucE | WP_011617409 | L-*erythro*-*β*-OH-Asp | R | (Kreutzer et al., 2012; Reitz et al., 2019) |
| | | CucF | WP_011617408 | L-*threo*-*β*-OH-Asp | S | (Kreutzer et al., 2012; Reitz et al., 2019) |
| Serobactin | *Herbaspirillum seropedicae* Z67 | SbtI1 | ADJ63842 | L-*threo*-*β*-OH-Asp | S | (Rosconi et al., 2013; Reitz et al., 2019) |
| | | SbtH | ADJ63841 | D-*threo*-*β*-OH-Asp | R | (Rosconi et al., 2013; Reitz et al., 2019) |
| Ornibactin | *Burkholderia cenocepacia* J2315 | OrbG | AUD12000 | D-*threo*-*β*-OH-Asp | R | (Stephan et al., 1993; Agnoli et al., 2006) |
| Delftibactin | *Delftia acidovorans* SPH-1 | DelD | ABX37386 | L-*erythro*-*β*-OH-Asp | R | (Johnston et al., 2013; Reitz et al., 2019) |
| Syringomycin | *Pseudomonas syringae* pv. *syringae* B301D | SyrP | AAY37651 | L-*threo*-*β*-OH-Asp | S | (Singh et al., 2008) |
| Ulleungmycin | *Streptomyces* sp. KCB13F003 | None | ATU31797 | D-*threo*-*β*-OH-Asn | R | (Son et al., 2017) |
| Kutzneride | *Kutzneria* sp. 744 | ktzO | ABV56595 | L-*threo*-*β*-OH-Glu | S | (Strieker et al., 2009) |
| | | ktzP | ABV56596 | L-erythro-*β*-OH-Glu | R | (Strieker et al., 2009) |
| Curacomycin | *Streptomyces curacoi* NBRC 12761 | None | KUM80511 | D-*β*-OH-Asn | unkown | (Kaweewan et al., 2017) |
| Laspartomycins | *Streptomyces viridochromogenes* ATCC 29814 | orf19 | AEF16019 | L-Asp | - | (Wang et al., 2011) |
| Malacidin A | Uncultured bacterium | MlcN | ARU08082 | L-*β*-OH-Asp | unkown | (Hover et al., 2018) |
| Friulimicin A | *Actinoplanes firuliensis* | RegB | CAD32906 | L-Asp or L-Asn | - | (Muller et al., 2007) |

# Table S4. Annotation of potashchelin biosynthesis genes

| Gene designation | *Halomonas sp*. MG34 gene no. | Protein Accession no. (GenBank) | Size(aa) | Proposed function |
|---|---|---|---|---|
| *orf1* | FRY77_09780 | NAO96300 | 362 | DUF1513 domain-containing protein |
| *orf2* | FRY77_09785 | NAO96301 | 550 | multidrug ABC transporter permease/ATP-binding protein |
| *orf3* | FRY77_09790 | NAO96302 | 262 | siderophore-iron reductase FhuF |
| *orf4* | FRY77_09795 | NAO96303 | 255 | ATP-binding cassette domain-containing protein |
| *orf5* | FRY77_09800 | NAO96304 | 265 | inositol monophosphatase |
| *orf6* | FRY77_09805 | NAO96305 | 523 | AAA family ATPase |
| *orf7* | FRY77_09810 | NAO96306 | 521 | AAA family ATPase |
| *orf8* | FRY77_09815 | NAO96307 | 724 | TonB-dependent siderophore receptor |
| *orf9* | FRY77_09820 | NAO96308 | 346 | ABC transporter substrate-binding protein |
| *orf10* | FRY77_09825 | NAO96309 | 726 | TonB-dependent siderophore receptor |
| *orf11* | FRY77_09830 | NAO96310 | 201 | RNA polymerase factor sigma-70 |
| *orf12* | FRY77_09835 | NAO96311 | 68 | DUF4880 domain-containing protein |
| *orf13* | FRY77_09840 | NAO96312 | 81 | MbtH family NRPS accessory protein |
| *orf14* | FRY77_09845 | NAO96313 | 246 | type-II thioesterase |
| *orf15* | FRY77_09850 | NAO96314 | 270 | 4'-phosphopantetheinyl transferase |
| *ptcA* | FRY77_09855 | NAO96315 | 333 | TauD/TfdA family dioxygenase |
| *ptcB* | FRY77_09860 | NAO96316 | 1533 | Loading module: CAL-PCP-C-TauD |
| *ptcC* | FRY77_09865 | NAO96317 | 1061 | NPRS: C-A-PCP |
| *ptcD* | FRY77_09870 | NAO96318 | 2169 | NPRS: C-A-PCP-C-A-PCP |
| *ptcE* | FRY77_09875 | NAO96319 | 2190 | NPRS: C-A-PCP-C-A-PCP |
| *ptcF* | FRY77_09880 | NAO96320 | 4574 | NPRS: C-A-PCP-C-A-PCP- C-A-PCP- C-A-PCP-TE |
| *orf16* | FRY77_09885 | n/a | 14 | IS630 family transposase |
| *orf17* | FRY77_09890 | NAO96321 | 309 | ABC transporter substrate-binding protein |
| *orf18* | FRY77_09895 | NAO96322 | 738 | TonB-dependent siderophore receptor |
| *orf19* | FRY77_09900 | NAO96323 | 317 | lipase |
| *orf20* | FRY77_09905 | NAO96324 | 431 | Lysine/ornithine N-monooxygenase |
| *orf21* | FRY77_09910 | NAO96325 | 387 | N-acetyltransferase |
| *orf22* | FRY77_09915 | NAO96326 | 548 | cyclic peptide export ABC transporter |
| *orf23* | FRY77_09920 | NAO96327 | 207 | cytochrome b |
| *orf24* | FRY77_09925 | NAO96328 | 314 | siderophore-interacting protein |
| *orf25* | FRY77_09930 | NAO96329 | 666 | Fe(3+)-hydroxamate ABC transporter permease FhuB |
| *orf26* | FRY77_09935 | NAO96330 | 262 | siderophore-iron reductase FhuF |
| *orf27* | FRY77_09940 | NAO96331 | 553 | multidrug ABC transporter permease/ATP-binding protein |

**Reference:**


Agnoli, K., Lowe, C.A., Farmer, K.L., Husnain, S.I., and Thomas, M.S. (2006). The ornibactin biosynthesis and transport genes of Burkholderia cenocepacia are regulated by an extracytoplasmic function sigma factor which is a part of the Fur regulon. *J Bacteriol* 188(10)**,** 3631-3644. doi: 10.1128/JB.188.10.3631-3644.2006.

Balibar, C.J., Vaillancourt, F.H., and Walsh, C.T. (2005). Generation of D amino acid residues in assembly of arthrofactin by dual condensation/epimerization domains. *Chemistry & biology* 12(11)**,** 1189-1200.

Bitzer, J., Grosse, T., Wang, L., Lang, S., Beil, W., and Zeeck, A. (2006). New aminophenoxazinones from a marine Halomonas sp.: fermentation, structure elucidation, and biological activity. *The Journal of antibiotics* 59(2)**,** 86-92. doi: 10.1038/ja.2006.12.

Hover, B.M., Kim, S.H., Katz, M., Charlop-Powers, Z., Owen, J.G., Ternei, M.A., et al. (2018). Culture-independent discovery of the malacidins as calcium-dependent antibiotics with activity against multidrug-resistant Gram-positive pathogens. *Nat Microbiol* 3(4)**,** 415-422. doi: 10.1038/s41564-018-0110-1.

Johnston, C.W., Wyatt, M.A., Li, X., Ibrahim, A., Shuster, J., Southam, G., et al. (2013). Gold biomineralization by a metallophore from a gold-associated microbe. *Nat Chem Biol* 9(4)**,** 241-243. doi: nchembio.1179 [pii]

10.1038/nchembio.1179.

Kaweewan, I., Komaki, H., Hemmi, H., and Kodani, S. (2017). Isolation and structure determination of new antibacterial peptide curacomycin based on genome mining. *Asian Journal of Organic Chemistry* 6(12)**,** 1838-1844.

Kreutzer, M.F., Kage, H., and Nett, M. (2012). Structure and biosynthetic assembly of cupriachelin, a photoreactive siderophore from the bioplastic producer Cupriavidus necator H16. *J Am Chem Soc* 134(11)**,** 5415-5422. doi: 10.1021/ja300620z.

Kreutzer, M.F., and Nett, M. (2012). Genomics-driven discovery of taiwachelin, a lipopeptide siderophore from Cupriavidus taiwanensis. *Org Biomol Chem* 10(47)**,** 9338-9343. doi: 10.1039/c2ob26296g.

Kumar, S., Stecher, G., and Tamura, K. (2016). MEGA7: Molecular Evolutionary Genetics Analysis Version 7.0 for Bigger Datasets. *Molecular biology and evolution* 33(7)**,** 1870-1874. doi: msw054 [pii]

10.1093/molbev/msw054.

Muller, C., Nolden, S., Gebhardt, P., Heinzelmann, E., Lange, C., Puk, O., et al. (2007). Sequencing and analysis of the biosynthetic gene cluster of the lipopeptide antibiotic Friulimicin in Actinoplanes friuliensis. *Antimicrob Agents Chemother* 51(3)**,** 1028-1037. doi: 10.1128/AAC.00942-06.

Palomo, S., Gonzalez, I., de la Cruz, M., Martin, J., Tormo, J.R., Anderson, M., et al. (2013). Sponge-derived Kocuria and Micrococcus spp. as sources of the new thiazolyl peptide antibiotic kocurin. *Mar Drugs* 11(4)**,** 1071-1086. doi: 10.3390/md11041071.

Reitz, Z.L., Hardy, C.D., Suk, J., Bouvet, J., and Butler, A. (2019). Genomic analysis of siderophore beta-hydroxylases reveals divergent stereocontrol and expands the condensation domain family. *Proc Natl Acad Sci U S A* 116(40), 19805-19814. doi: 10.1073/pnas.1903161116.

Rosconi, F., Davyt, D., Martinez, V., Martinez, M., Abin-Carriquiry, J.A., Zane, H., et al. (2013). Identification and structural characterization of serobactins, a suite of lipopeptide siderophores produced by the grass endophyte Herbaspirillum seropedicae. *Environ Microbiol* 15(3), 916-927. doi: 10.1111/1462-2920.12075.

Saitou, N., and Nei, M. (1987). The neighbor-joining method: a new method for reconstructing phylogenetic trees. *Molecular biology and evolution* 4(4), 406-425.

Scholz-Schroeder, B.K., Soule, J.D., and Gross, D.C. (2003). The sypA, sypS, and sypC synthetase genes encode twenty-two modules involved in the nonribosomal peptide synthesis of syringopeptin by Pseudomonas syringae pv. syringae B301D. *Mol Plant Microbe Interact* 16(4), 271-280. doi: 10.1094/MPMI.2003.16.4.271.

Shirling, E.T., and Gottlieb, D. (1966). Methods for characterization of Streptomyces species. *International journal of systematic bacteriology* 16(3), 313-340.

Singh, G.M., Fortin, P.D., Koglin, A., and Walsh, C.T. (2008). beta-Hydroxylation of the aspartyl residue in the phytotoxin syringomycin E: characterization of two candidate hydroxylases AspH and SyrP in Pseudomonas syringae. *Biochemistry* 47(43), 11310-11320. doi: 10.1021/bi801322z.

Son, S., Hong, Y.S., Jang, M., Heo, K.T., Lee, B., Jang, J.P., et al. (2017). Genomics-Driven Discovery of Chlorinated Cyclic Hexapeptides Ulleungmycins A and B from a Streptomyces Species. *J Nat Prod* 80(11), 3025-3031. doi: 10.1021/acs.jnatprod.7b00660.

Stephan, H., Freund, S., Beck, W., Jung, G., Meyer, J.M., and Winkelmann, G. (1993). Ornibactins--a new family of siderophores from Pseudomonas. *Biometals* 6(2), 93-100. doi: 10.1007/bf00140109.

Strieker, M., Nolan, E.M., Walsh, C.T., and Marahiel, M.A. (2009). Stereospecific synthesis of threo- and erythro-beta-hydroxyglutamic acid during kutzneride biosynthesis. *J Am Chem Soc* 131(37), 13523-13530. doi: 10.1021/ja9054417.

Wang, Y., Chen, Y., Shen, Q., and Yin, X. (2011). Molecular cloning and identification of the laspartomycin biosynthetic gene cluster from Streptomyces viridochromogenes. *Gene* 483(1-2), 11-21. doi: 10.1016/j.gene.2011.05.005.
